# What is the ideal glucose range for a patient with sepsis in the ICU? A retrospective analysis of MIMIC-IV

**DOI:** 10.1136/bmjopen-2025-104916

**Published:** 2026-01-28

**Authors:** Tristan Struja, Lasse Hyldig Hansen, João Matos, Josep Gómez, Alex Pardo, Ismini Lourentzou, Nima Hejazi, Leo Anthony Celi, Andre Kurepa Waschka

**Affiliations:** 1 Medical University Clinic, Kantonsspital Aarau, Aarau, Switzerland; 2 IMES, Labor for Computational Physiology, Massachusetts Institute of Technology, Cambridge, Massachusetts, USA; 3 Jens Chr. Skou 2, Aarhus University, Aarhus, Denmark; 4 Faculty of Engineering, University of Porto, Porto, Portugal; 5 Hospital Universitari de Tarragona Joan XXIII, Tarragona, Spain; 6 School of Information Sciences, University of Illinois Urbana-Champaign, Urbana, Illinois, USA; 7 Department of Biostatistics, Harvard University, Cambridge, Massachusetts, USA; 8 Mathematics & Statistics, Elon University, Elon, North Carolina, USA

**Keywords:** Sepsis, INTENSIVE & CRITICAL CARE, DIABETES & ENDOCRINOLOGY

## Abstract

**Abstract:**

**Importance:**

Clinical trials have produced inconclusive results regarding the optimal glucose range for a patient with sepsis in the intensive care unit (ICU) receiving insulin treatment.

**Objective:**

To investigate the optimal glucose range in patients with sepsis in the ICU independent of confounding covariates.

**Design:**

Targeted trial emulation of glucose ranges using causal inference targeted maximum likelihood estimation and longitudinal mixed-effects models combined with survival models.

**Setting:**

Single-centre, academic referral hospital in Boston, Massachusetts, USA.

**Participants:**

Adults fulfilling sepsis 3 criteria with at least three glucose readings and insulin treatment from the Medical Information Mart for Intensive Care (MIMIC)-IV database (2008–2019).

**Exposure:**

Five predefined glucose distributions with means at 100, 130, 160 (baseline), 190 and 220 mg/dL mimicking current guidelines’ recommendations (140–180 mg/dL).

**Main outcome and measure:**

The primary outcome was in-hospital mortality. Modified counterfactual treatment-policy risks across distinct time-weighted glucose ranges were estimated.

**Results:**

Of 73 181 eligible patients, 8002 patients with a median age of 66 years (41% women, 67% white ethnicity, 57% diabetes) were included. There was a U-shaped curve between glucose range and mortality in patients without diabetes, but overall, this association was not significant (mean glucose at 100 mg/dL with 21% mortality and mean glucose at 220 mg/dL with 26% mortality, p-for-trend 0.26). Mortality was lowest at 17%, with mean glucose between 130 and 160 mg/dL. Hypoglycaemic events (<80 mg/dL) became increasingly more frequent with tighter glucose control 16% at 220 mg/dL compared with 77% at 100 mg/dL (p-for-trend 0.01). Joint modelling corroborated these results and did not identify covariates that would favour lower glucose ranges in subsets of patients.

**Conclusion and relevance:**

Our data suggest a U-shaped association of glucose and mortality with an optimal average glucose between 160 and 190 mg/dL. These results confirm current guideline recommendations. Together with recent results from randomised controlled trials, intensivists should aim for a liberal glucose range in most patients.

**STRENGTHS AND LIMITATIONS OF THIS STUDY**

- High-resolution electronic intensive care unit (ICU) data from the MIMIC-IV database enabled time-updated adjustment for a large set of clinical covariates.
- The single-centre design limits generalisability, as the cohort may not reflect the demographic and clinical characteristics of broader ICU populations.
- This study used a targeted trial emulation framework with doubly robust targeted maximum likelihood estimation (TMLE) to reduce bias from model misspecification.
- The combination of TMLE with joint longitudinal–survival modelling allowed assessment of both causal effects and glycaemic variability.
- As with all observational studies, causal inference relies on the assumption that all relevant confounders were measured and appropriately adjusted for.

## Introduction

 In critically ill patients admitted to the intensive care unit (ICU), especially in those with sepsis, dysglycaemia and high variability of glucose are common occurrences and are associated with unfavourable outcomes.^1^,^4^ However, it is a matter of debate whether this link implies causation, as randomised controlled trials have yielded varying results when it comes to lowering blood glucose.^5^,^9^

Notably, previous single-centre trials with atypical patient populations indicated that lowering blood glucose with insulin to a healthy, age-adjusted fasting range resulted in improved outcomes, compared with permissive hyperglycaemia.^6 10^ On the other hand, larger multicentre trials did not confirm the benefits of tight glucose control, and the largest of these reported increased mortality,^9^ which was linked to a significantly higher occurrence of hypoglycaemia (glucose<80 mg/dL).^11^

These contradictory results may be due to two key methodological differences such as inconsistent blood glucose measurements and insulin adjustments potentially leading to hypoglycaemia.^12^ Some have also questioned the benefit observed in earlier trials employing early use of parenteral nutrition,^6 10 13^ as subsequent research indicated that this may raise the risk of infections and delay recovery from critical illness.^14^ To fill this knowledge gap, Gunst *et al* conducted a pivotal multicentre randomised controlled trial (RCT) in a general ICU cohort.^5^ Despite randomising 9230 patients to either tight glucose control (glucose target range of 80–110 mg/dL) or liberal control (insulin initiated only when the blood glucose level was >215 mg/dL with a target range of 180–215 mg/dL), the authors were not able to show significant differences in important clinical outcomes such as ICU length of stay (LOS), 90-day mortality, nosocomial infections or in-hospital mortality between the groups.

Estimating clinical effectiveness of interventions has become more feasible and precise with the advent of advanced analytical frameworks that leverage the power of machine learning technologies. Combining two approaches can further solidify conclusions. While targeted maximum likelihood estimation (TMLE) is a semiparametric causal inference framework estimating an average treatment effect adjusting for a range of confounding factors, it cannot account for changes over time.^15^ Here, joint modelling can simultaneously incorporate information from longitudinal mixed-effect models into a time-to-event model capturing the crucial and dynamic interplay of changing patient conditions, such as fluctuating glucose levels.^16^

With this combination, finding an optimal glucose range for patients with sepsis using real-world data from high-resolution datasets such as Medical Information Mart for Intensive Care (MIMIC)-IV becomes possible^17^; a task which could be too costly, impractical or even unethical for an RCT. We hypothesised that baseline covariates might predict which patients with sepsis in the ICU might benefit from lower glucose ranges.

## Methods

This study is reported in accordance with the Strengthening the Reporting of Observational studies in Epidemiology statement.^18^ Data were extracted in 2023 from the open-access and de-identified MIMIC-IV V.2.2 (2008–2019) using Google’s BigQuery software. MIMIC-IV is maintained by the Laboratory for Computational Physiology at Massachusetts Institute of Technology (MIT).^19^ During this time frame, there were no standardised sepsis protocols in place at Beth Israel Deaconess Medical Center. MIMIC-IV includes physiological data collected from bedside monitors, as well as other clinical variables and provider documents. Approximately 70 000 de-identified medical ICU records are archived in MIMIC-IV.

### Cohort selection

All patients older than 18 years of age who had sepsis as defined by the sepsis 3 criteria were included in the analysis.^20^ We only included first-time ICU stays and excluded cases with an ICU LOS of <2 days or <3 glucose measurements. To avoid immortal time bias, treatment assignment was only possible during an eligibility period of the first 24 hours for insulin treatment. Immortal time bias can lead to erroneous conclusions about the effectiveness of treatments or interventions if not properly accounted for in the study design and analysis.^21^ If treatment was initiated after this eligibility period, the patient was retained in the control group, emulating a targeted trial.^22^ Diabetes was assigned by hospital billing codes (see online supplemental table 1). The baseline time-weighted average glucose of our cohort was 160 mg/dL. We then shifted this distribution to new, predefined glucose means of 100, 130, 190 and 220 mg/dL (see online supplemental figure 2) mimicking current guidelines’ recommendations of a target range of 140–180 mg/dL.^4 23^

### Covariates

Patient-level variables were obtained from the database at the time of ICU admission and time-varying variables were aggregated by calculating the time-weighted average for the TMLE and survival analyses (eg, sequential organ failure assessment (SOFA) score burden was calculated for every hour in the ICU); in case of missing values, the last observation was carried forward and an hourly weighted average for the whole stay was calculated (see online supplemental table 1). For joint modelling, we extracted time-varying variables at hourly intervals of glucose measurements (see online supplemental table 1). We used closest-neighbour matching for other time-varying variables. We further extracted International Classification of Diseases, Tenth Revision codes for key comorbidities.

### Outcomes

The primary outcome was in-hospital mortality, including discharge to hospice care. Secondary outcomes were hospital-free days and ICU-free days by day 28, with deceased patients being assigned zero free days, SOFA burden and combined nosocomial infections (central-line associated bloodstream infection, catheter-associated urinary tract infection, surgical site infection, ventilator-associated pneumonia). The occurrence of hypoglycaemia was the primary safety outcome (<50 and <80 mg/dL). We used the time of discharge or death at an odd versus an even hour as a negative control outcome, as treatment should not affect this random event.^24^

### Statistical analysis

Statistical analyses were performed using R V.4.3.1^25^ and Python V.3.10.^26^ We applied two complementary analytical frameworks: (1) TMLE to estimate causal effects of glucose shifts on clinical outcomes and (2) joint longitudinal–survival modelling to examine associations between glycaemic trajectories, organ dysfunction as per SOFA score and mortality.

For causal estimation, we used TMLE implemented in the tmle3 package (V.0.2). In this framework, the exposure (time-weighted mean glucose) is conceptualised as the treatment variable.^15^ We estimated a modified counterfactual treatment-policy risk (MTP, or more loosely the shifted average treatment effect), defined as counterfactual shifts of the observed glucose distribution by prespecified amounts while preserving its shape. These yield marginal, population-level effect estimates comparing outcomes under shifted versus unshifted glucose distributions. Nuisance functions, that is, the outcome regression and treatment mechanism, were estimated using the SuperLearner3 ensemble algorithm with fivefold cross-validation. TMLE relies on standard causal assumptions, including conditional exchangeability and positivity. We examined treatment feasibility across glucose strata and tabulated covariates by exposure to assess potential violations. Because TMLE provides population-average effects but does not yield insight into covariate-specific heterogeneity, additional modelling was pursued.

To evaluate glucose trajectories and explore whether patient characteristics modified the association between glycaemic patterns and outcomes, we fitted joint longitudinal–survival models using the JMBayes2 package (V.0.4.5).^27^ This approach simultaneously links longitudinal biomarker trajectories with a time-to-event model. We estimated two linear mixed-effects submodels: one for SOFA scores and one for insulin dosing, with hourly glucose values and other confounders included as predictors. We incorporated random intercepts per admission SOFA value and per hour and random slopes for each patient to capture individual trajectories over time. The longitudinal processes were then linked to a Cox proportional hazards survival model for death or discharge, allowing a flexible, non-constant association between evolving markers and the event risk.^16^ All continuous covariates were mean-centred and modelled using natural cubic splines with five knots (see online supplemental table 2 for model specifications). Bayesian estimation was conducted via Markov chain Monte Carlo using five chains with 16 000 iterations each, including a burn-in of 4000 iterations, and employing non-informative priors. Convergence was assessed through trace plots.

Given the exploratory, hypothesis-generating nature of this work, we did not compute p values and instead report 95% CIs (TMLE models) and 95% credible intervals (joint models).

### Patient and public involvement

None.

## Results

**Figure 1:**
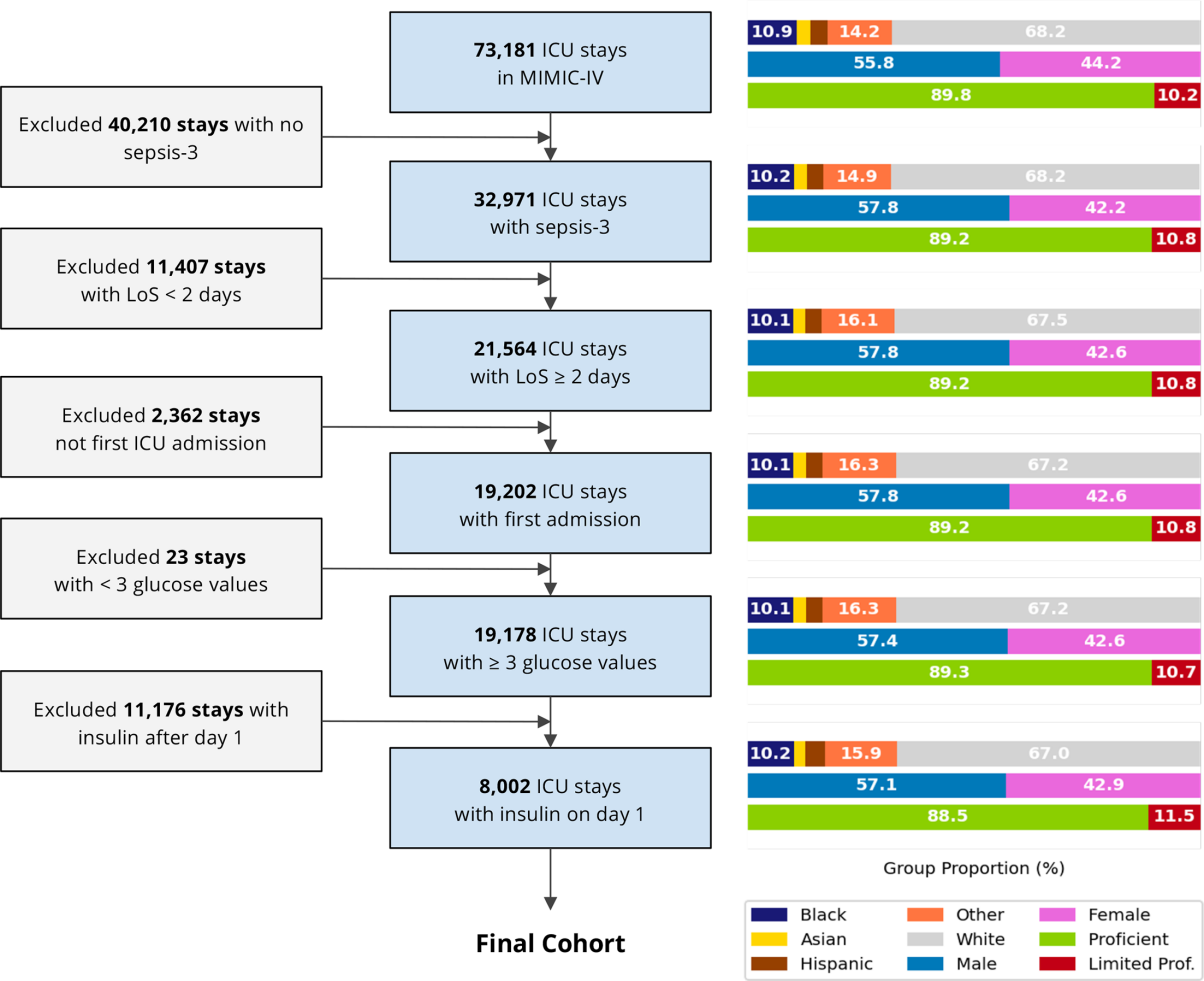
Study cohort selection flow chart with composition of key demographic variables. ICU, intensive care unit; Limited Prof., limited English proficiency; LOS, length of stay; MIMIC-IV, Medical Information Mart for Intensive Care-IV.

After selecting our cohort, we had a sample size of 8002 admissions (see figure 1). Table 1 depicts the baseline characteristics of our cohort stratified by diabetes status (for additional data, see online supplemental table 3). Median age was 66 years, 40.8% were women and 67% were of white ethnicity. Median ICU LOS was 5.7 days in case a patient died and 4.1 days if patients survived. 17.2% of patients admitted to an ICU died or were discharged to a hospice (1.7% of all discharges, data not shown). Baseline Charlson comorbidity index, age, body mass index and admission SOFA scores between patients with diabetes and patients without diabetes were evenly balanced. However, there were marked differences in the use of renal replacement therapy (RRT) (17.1% in the diabetes group, 11.4% in the non-diabetes group); in the use of invasive mechanical ventilation (IMV) (60.8% vs 76.1%, respectively); in the use of vasopressors (VP) (57.1% vs 72.9%, respectively); in the prevalence of hypertension; chronic kidney disease and time-weighted glucose (177 vs 139 mg/dL, respectively). Both groups had a non-zero probability of having time-weighted glucose within the studied treatment range (100–220 mg/dL), which gives no concerns for violation of the positivity assumption, except for values well in the hypoglycaemic range <80 mg/dL.

**Table 1:** Baseline information on the study cohort

	Diabetes absent (N=3418)	Diabetes present (N=4584)	Overall (N=8002)
In-hospital mortality	543 (15.9%)	834 (18.2%)	1377 (17.2%)
ICU LOS if died (days), median (IQR)	5.54 (3.58, 10.9)	5.79 (3.58, 11.2)	5.71 (3.58, 11.1)
ICU LOS if survived (days), median (IQR)	4.17 (2.67, 8.04)	4.04 (2.79, 7.28)	4.13 (2.75, 7.63)
Invasive mechanical ventilation	2602 (76.1%)	2788 (60.8%)	5390 (67.4%)
Renal replacement therapy	390 (11.4%)	786 (17.1%)	1176 (14.7%)
Vasopressor(s)	2493 (72.9%)	2617 (57.1%)	5110 (63.9%)
Age overall (years), median (IQR)	66.0 (56.0, 77.0)	66.0 (58.0, 75.0)	66.0 (57.0, 76.0)
Sex female	1360 (39.8%)	1905 (41.6%)	3265 (40.8%)
Race			
Asian	93 (2.7%)	117 (2.6%)	210 (2.6%)
Black	217 (6.3%)	600 (13.1%)	817 (10.2%)
Hispanic	112 (3.3%)	234 (5.1%)	346 (4.3%)
Other	576 (16.9%)	694 (15.1%)	1270 (15.9%)
White	2420 (70.8%)	2939 (64.1%)	5359 (67.0%)
Admission SOFA score, median (IQR)	6.00 (4.00–9.00)	6.00 (4.00–9.00)	6.00 (4.00–9.00)
Charlson comorbidity index, median (IQR)	5.00 (4.00–7.00)	7.00 (6.00–9.00)	6.00 (4.00–8.00)
BMI (kg/m^2^), median (IQR)	27.2 (23.8–31.4)	29.6 (25.2–34.9)	28.4 (24.4–33.5)
TW average SOFA score, mean (SD)	5.44 (2.71)	5.23 (2.62)	5.32 (2.66)
TW average glucose (mg/dl), mean (SD)	139 (27.6)	177 (41.6)	161 (40.9)
TW average glucose (mg/dL)			
≤80	0 (0%)	1 (0.0%)	1 (0.0%)
81–110	177 (5.2%)	57 (1.2%)	234 (2.9%)
111–140	2048 (59.9%)	834 (18.2%)	2882 (36.0%)
141–180	930 (27.2%)	1749 (38.2%)	2679 (33.5%)
181–240	229 (6.7%)	1569 (34.2%)	1798 (22.5%)
≥241	34 (1.0%)	374 (8.2%)	408 (5.1%)
Parenteral nutrition given	182 (5.3%)	148 (3.2%)	330 (4.1%)
Enteral nutrition given	960 (28.1%)	1267 (27.6%)	2227 (27.8%)
TW average insulin (units), mean (SD)	2.67 (5.01)	3.34 (2.91)	3.06 (3.96)

BMI, body mass index; ICU, intensive care unit; LOS, length of stay; SOFA, sequential organ failure assessment; TW, time-weighted.

### TMLE: negative control outcome

**Figure 2:**
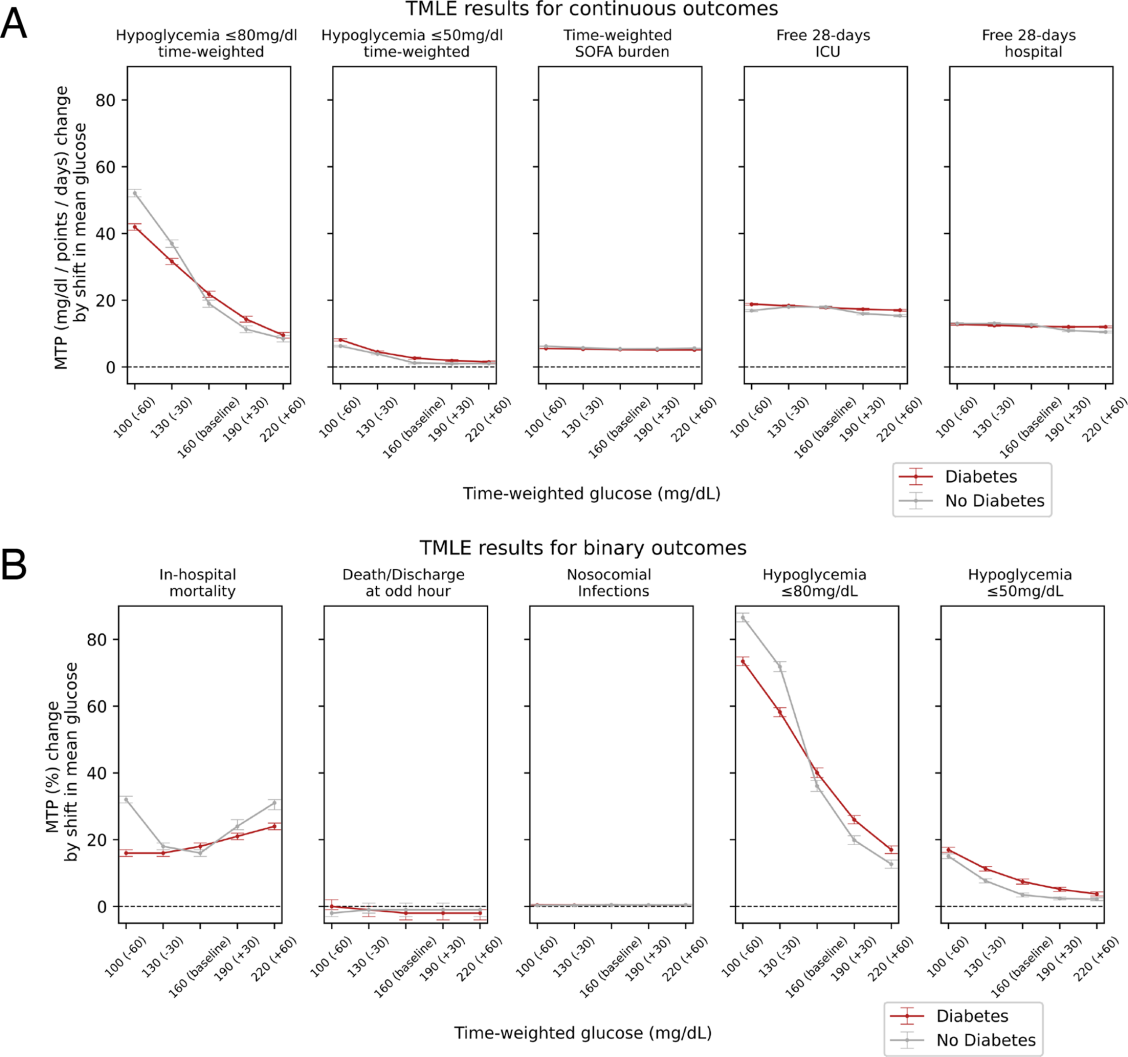
TMLE-derived average treatment effects over the five strata of mean glucose; (**A**) binary outcomes; (**B**) continuous outcomes. Stratified by diabetes status of patients. ICU, intensive care unit; MTP, modified counterfactual treatment-policy risk; TMLE, targeted maximum likelihood.

Assuming that death or discharge should occur at random times, we binarised these events to odd versus even hours of the day. In adjusted TMLE models, there was no distinct treatment effect in any glucose stratum (see figure 2 and table 2), suggesting that residual confounding is negligible.^21^

**Table 2:** TMLE results not stratified by diabetes status (n=8002)

TMLE model (MTP (95% CI))							
MTP (95% CI)	Glucose mean (shift, IQR)	100 mg/dL (−60 mg/dL)	130 mg/dL (−30 mg/dL)	160 mg/dL (baseline)	190 mg/dL (+30 mg/dL)	220 mg/dL (+60 mg/dL)	P for trend*
Mortality		21% (20% to 22%)	17% (16% to 17%)	17% (16% to 18%)	22% (21% to 23%)	26% (25% to 26%)	0.26
Odd versus even hour		49% (48% to 50%)	49% (48% to 50%)	48% (47% to 49%)	48% (47% to 49%)	48% (47% to 49%)	0.06
Hypoglycaemia mild<80 mg/dL		77% (77% to 78%)	63% (62% to 64%)	38% (37% to 39%)	24% (23% to 25%)	16% (15% to 16%)	0.01
Hypoglycaemia severe<50 mg/dL		17% (17% to 18%)	10% (10% to 11%)	6% (5% to 6%)	4% (4% to 4%)	3% (3% to 3%)	0.02
Nosocomial infection		0.4% (0.3% to 0.5%)	0.4% (0.3% to 0.5%)	0.5% (0.3% to 0.6%)	0.4% (0.3% to 0.6%)	0.4% (0.3% to 0.6%)	1.00
Time-weighted SOFA burden		6 (6 to 6)	6 (5 to 6)	5 (5 to 5)	5 (5 to 5)	5 (5 to 5)	0.06
28 ICU-free days		19 (18 to 19)	18 (18 to 19)	18 (18 to 18)	17 (17 to 17)	17 (16 to 17)	0.06
28 hospital-free days		13 (13 to 13)	13 (13 to 13)	12 (12 to 13)	12 (12 to 12)	12 (11 to 12)	0.02

* P for trend calculated with Pearson’s correlation coefficient and t-statistic.

ICU, intensive care unit; MTP, modified counterfactual treatment policy risk; SOFA, sequential organ failure assessment score; TMLE, targeted maximum likelihood estimation.

### TMLE: primary and secondary outcomes

**Figure 3:**
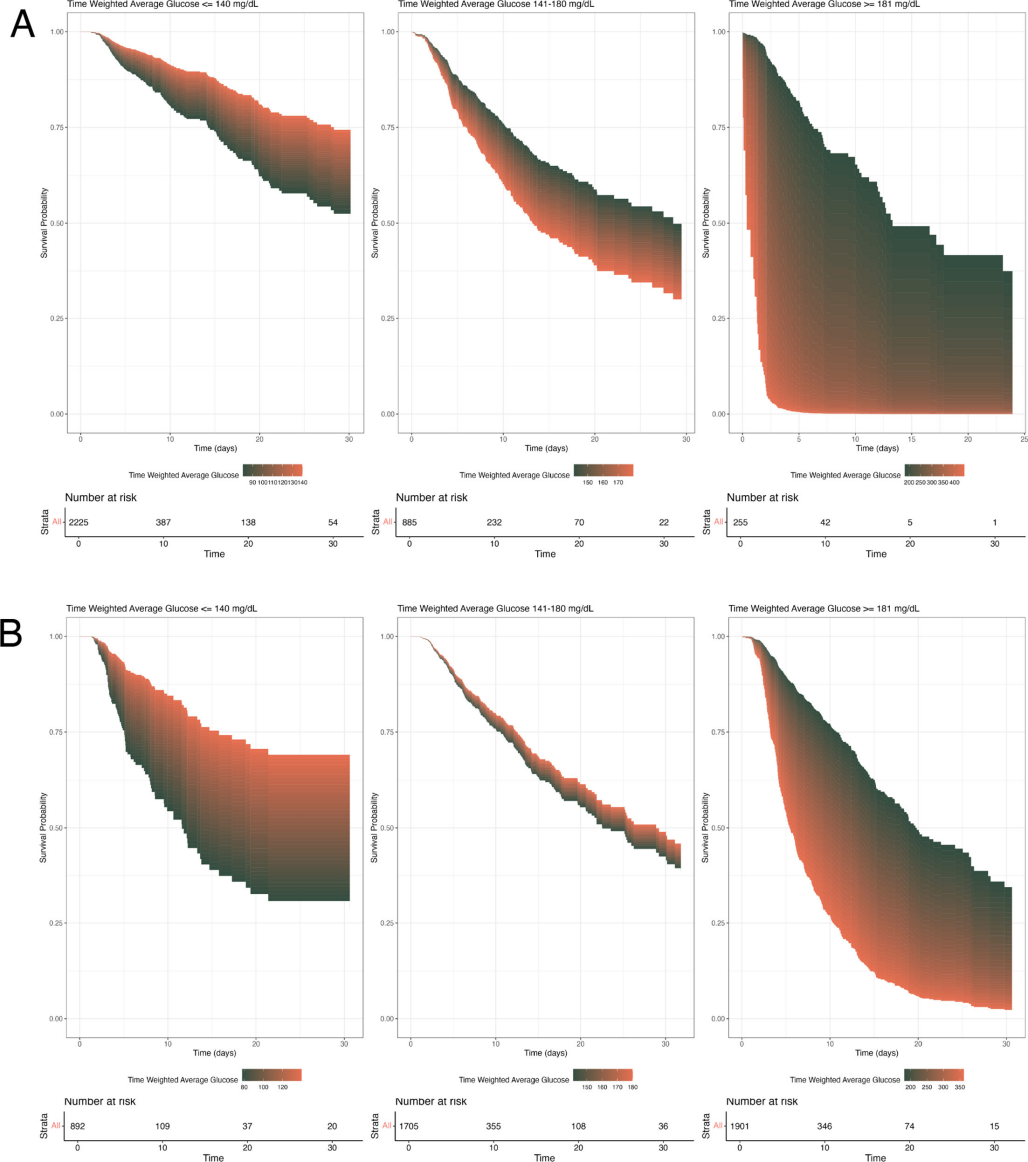
Cox model-derived survival curves split by time-weighted average glucose into bins matching current guidelines’ suggestions; (**A**) patients without diabetes; (**B**) patients with diabetes.

There was no clear increase or decrease in in-hospital mortality over all glucose strata with mean glucose at 100 mg/dL (mean shift −60 mg/dL, MTP mortality 21% (95% CI 20% to 22%)), 130 mg/dL (−30 mg/dL, MTP 17% (95% CI 16% to 18%)), 160 mg/dL (baseline, MTP 17% (95% CI 16% to 17%)), 190 mg/dL (+30 mg/dL, MTP 22% (95% CI 21% to 23%)) or 220 mg/dL (+60 mg/dL, MTP 26% (95% CI 25% to 26%)) (see figure 2, table 2 and online supplemental figure 2). As expected, the probability of both a mild or severe hypoglycaemic event increased with lower glucose target ranges irrespective of concomitant diabetes. This effect was also observed when using the continuous time-weighted glucose hypoglycaemia ranges (see figure 3B). Interestingly, there was a trend for more 28 hospital-free days and 28 ICU-free days with lower glucose target ranges. When using time-weighted SOFA scores as an outcome of illness severity over time, it did not observe changes in illness severity over time with different glucose target ranges. All these causal effects were observed irrespective of diabetes status or when the diabetes and no-diabetes groups were combined (see online supplemental figure 1).

### Joint modelling: negative control outcome

We fitted an adjusted Cox model with the outcome of death or discharge at odd versus even hours of the day. When plotting the two survival curves of people who died versus those who survived, there was no distinct difference between the survival curves (see online supplemental figure 3), suggesting that residual confounding is negligible.^21^ Also, we saw no clear violation of the proportional hazards assumption when plotting the Schoenfeld residuals over time (see online supplemental figure 4).

### Joint modelling: primary and secondary outcomes

We plotted survival curves corresponding to the time-weighted glucose stratified by diabetes status displaying a heterogeneous association (see figure 3). Patients with a time-weighted glucose between 141 and 180 mg/dL, especially in those with diabetes, closely mirroring current guidelines, had no discernible treatment effect. However, patients below 140 mg/dL had the highest survival probability of the three groups, but showed increasing mortality with lower glucose values more pronounced in patients with diabetes, while patients over 180 mg/dL showed an increased mortality especially in patients with diabetes (see figure 2B) compared with patients without diabetes (see figure 2A). In the longitudinal model with hourly SOFA as the outcome (see online supplemental table 4, outcome hourly SOFA), most relevant predictors of higher SOFA values were invasive treatments such as IMV (β 0.3 (95% CR 0.2 to 0.3)), RRT (β 0.8 (95% CR 0.7 to 0.8)) and vasopressors (β 0.4 (95% CR 0.4 to 0.5)), and higher Charlson index scores (β range 0.3−0.8 (spline 1−5)), while diabetes (β −0.2 (95% CR −0.2 to −0.1)), elective admission (β −0.1 (95% CR −0.1 to 0.0)) and surgery (β −0.1 (95% CR −0.2 to −0.1)) were identified as protective factors. Hourly glucose itself was not predictive of hourly SOFA (β range −0.3 to 0.4 (spline 1−5)).

In the longitudinal model with hourly insulin dose as the outcome (see online supplemental table 4), most relevant predictors of more intense insulin treatment were hourly glucose (β range 0.4−2.0 (spline 1−5)), higher time-weighted average of glucose (β 0.2 (95% CR 0.2 to 0.2)), diabetes (β 0.1 (95% CR 0.1 to 0.1)) and surgery (β 0.3 (95% CR 0.3 to 0.3)), while missing glucose readings (β −0.1 (95% CR −0.1 to −0.1)) and a higher BMI (β range −9.8 to 2.6 (spline 1−5)) were associated with less hourly insulin.

In the survival model with mortality as the outcome (see online supplemental table 5), the most influential predictors were IMV (HR 0.8 (95% CR 0.7 to 0.9)), RRT (HR 0.6 (95% CR 0.5 to 0.6)), major surgery (HR 0.6 (95% CR 0.5 to 0.7)), elective admission (HR 0.4 (95% CR 0.3 to 0.6)) and English proficiency (HR 1.5 (95% CR 1.2 to 1.7)). Notably, patients with diabetes had lower hazards of dying (HR 0.7 (95% CR 0.6 to 0.8)). In joint modelling, the association parameters represent the relationship between the longitudinal outcome and the time-to-event outcome. In our case, the association parameter for the slope of the hourly insulin dose for the last 24 hours was highly influential (HR<0.01 (95% CR<0.01 to <0.01)), indicating that changes in the hourly insulin dose seem to be a mere proxy of treatment decisions before death or discharge. The average SOFA score over the last 24 hours was a significant predictor of mortality (HR 2.3 (95% CR 2.1 to 2.4)), suggesting that an increase in the SOFA score is associated with an increased risk of mortality.

The survival models with hypoglycaemia as the outcome showed similar effect sizes of the predictors except for diabetes, administration of carbohydrates, SOFA at admission and hourly SOFA.

## Discussion

Using a causal inference framework in a large cohort of critically ill patients with sepsis, we found no evidence that tighter glucose control with higher insulin exposure improved any clinically meaningful outcome. Across all TMLE analyses, glucose-lowering was not associated with reduced mortality, reduced SOFA burden, fewer nosocomial infections or more hospital-free or ICU-free days. Instead, lower glucose ranges consistently increased the risk of hypoglycaemia. An average glucose between 160 and 190 mg/dL seemed to be the optimal compromise. These findings were corroborated by joint modelling, which integrated hourly glucose values and patient-specific trajectories, but was not able to identify a covariate pattern or subgroup that benefited from lower glucose ranges.

Causal inference on high-resolution electronic health record data offers a robust method of detecting test and treatment disparities that are not explained by clinical factors and, if present, quantifying their effect sizes on patient outcomes. TMLE is a semiparametric estimator based on machine learning algorithms to minimise the risk of model misspecification.^28^ As TMLE is doubly robust, if either the outcome regression or the exposure mechanism is consistently estimated, it will yield unbiased estimates. Additionally, its estimates will always stay within the bounds of the original outcome, thus making it more robust to outliers and spare data. Finally, since TMLE fully incorporates machine learning, it is also a very attractive choice for analysing complex observational data with a large number of variables and potentially complex relationships.^28^ Valid interpretation of our estimates relies on several core assumptions of the causal inference framework as outlined by Hernán and Robins.^29^ These include exchangeability (ie, absence of unmeasured confounding after adjustment), positivity (ie, non-zero probability of receiving each treatment level within covariate strata), consistency (ie, well-defined and comparable exposure interventions) and adequate model specification. Although TMLE mitigates model misspecification through data-adaptive estimation, we acknowledge that the assumption of no unmeasured confounding can hardly be ever verified in any observational ICU research. Consequently, our findings should be interpreted as causally suggestive rather than definitive.

While TMLE used time-weighted averages, joint modelling used the full hourly glucose series, capturing both within-patient variability and time-dependent confounding. The fact that both methods provided similar results despite their conceptual and statistical differences strengthens our core finding that lowering glucose more aggressively did not meaningfully improve outcomes in patients with sepsis in the ICU. By integrating TMLE’s causal inference capabilities with the longitudinal data analysis strengths of joint modelling, our study provides a more comprehensive understanding of the optimal glucose management strategies for individual patients with sepsis in the ICU, allowing us to account for the diverse and dynamic treatment decisions during a patient’s ICU stay. Although mixed-effects or generalised additive models-based approaches could be used descriptively, our joint modelling framework already incorporates the longitudinal correlation structure.

Interestingly, our analysis found that tighter glucose control was not associated with relevant improvement in clinically meaningful outcomes, neither for patients with or without diabetes, but conferred a higher risk of adverse outcomes such as hypoglycaemia. Our negative results are likely explained by the fact that any potential benefit of lower glucose levels is counteracted by insulin-induced glycaemic variability and hypoglycaemia, both of which are independent predictors of mortality in sepsis.^2 12^ Moreover, stress hyperglycaemia in sepsis may represent a partly adaptive metabolic response, particularly in non-diabetic patients, which could explain why lower glucose ranges did not translate into improved outcomes.^4^ This is in line with several previous studies, most recently with the study by Gunst *et al*.^5^ Our treatment ranges were also in the range of 140–180 mg/dL proposed by current guidelines.^23^

Numerous studies have found that various interventions in the ICU are not beneficial, or even harmful, and a ‘less is more’ strategy may be favourable in critical care.^30^ Besides doing less in daily practice, it might also be time to further drive the use of large-scale, high-resolution databases and causal inference frameworks to answer pertinent clinical questions instead of relying on RCTs. Over the last two decades, 21 409 patients were randomised in costly glucose-lowering ICU trials and potentially exposed to unnecessary harm.^12^ New technologies can help reduce the need for clinical trials by paving the ground in observational data. Once potential avenues have been thoroughly explored, fewer RCTs will suffice to answer the clinical question.^22 31^

The survival curves for the three distinct glucose ranges add a dimension to our understanding of glucose management in patients with sepsis in the ICU. Particularly, the curves suggest that glucose levels ≤140 mg/dL in patients with diabetes especially might be harmful, as they are associated with a rapid decline in survival, echoing the risk of hypoglycaemia highlighted in our TMLE models, whereas this effect is less pronounced in patients without diabetes. For glucose levels between 141 and 180 mg/dL, the survival curves indicate a plateau effect, which corresponds with currently recommended glucose management guidelines, and this may reflect a more optimal glucose target range for patients with sepsis in the ICU. At glucose levels ≥181 mg/dL, there is a marked negative impact of high glucose levels on survival, especially for those without diabetes which has been well-documented in previous research.^23^

### Limitations

The main limitation of our analysis is that we only have data from one academic centre in the USA. We note that our cohort differs substantially from the general population of Massachusetts. State census data indicate a median age of ~40 years^32^ and a prevalence of diagnosed diabetes around 9% among adults^33^ compared with a median age of 66 years and 57% diabetes prevalence in our sample. On the other hand, race ethnicity of the cohort was very similar to census data, where roughly 69% of Massachusetts’ residents identify as white. As such, our cohort likely represents a medically complex, older and more comorbid population than community-based samples, which limits generalisability.

Time-varying covariates had missing values handled via last observation carried forward or assumed absence, which may introduce bias if missingness was not random. Missingness in large observational ICU databases such as MIMIC-IV is complex and often driven by clinical workflow, severity of illness and provider judgement rather than random processes, as demonstrated in prior work.^34^,^38^ For physiological monitor-derived variables, missingness may approximate missingness at random, but for laboratory measurements, medications and interventions, accumulating evidence indicates predominantly missingness not at random patterns. Our own recent analyses of lactate^39^ and glucose monitoring^40^ using MIMIC-IV data further support that measurement frequency reflects clinical acuity rather than demographic factors alone. Because standard imputation methods generally rely on missingness at random assumptions, alternative imputation strategies may introduce additional bias rather than mitigate it. Additionally, previous studies, including the recent SOFA-2 score development^41^ and a Massachusetts-based ICU analysis,^42^ suggest that alternative imputation methods do not substantially alter results in such settings. Reassuringly, we incorporated an imputation indicator for glucose measurement in the joint model and used two distinct analytic frameworks that yielded concordant results, but naturally residual bias from missing data mechanisms can never be fully excluded.

Resources and protocols can vary across healthcare providers and thus add an important layer to the generalisability of every study. For instance, detailed institutional insulin-infusion protocols and glucose-monitoring frequency from Beth Israel Deaconess Medical Center are not publicly available. Hence, we cannot exclude that variation in dosing adjustments, measurement intervals or protocol adherence contributed to the observed hypoglycaemic episodes. However, an analysis at the aforementioned institution found heterogeneous insulin treatment strategies vary significantly, irrespective of blood glucose level or diabetic status.^34^ Even though other publicly available ICU databases exist, they either lack the high-resolution in data points of MIMIC-IV (eg, eICU-Collaborative Research Database) or do not provide essential diagnoses and demographics although having a higher resolution in data points (eg, AmsterdamUMCdb, the high-time resolution ICU database (HiRID)).^43^

Although our sample size was similar to concurrent RCTs, our sample size might be insufficient to detect smaller benefits, especially given the narrowing support of data on both tails of the distribution. But even if such benefits exist, they need to be weighed against the clear and steep increase in risk for hypoglycaemia.

## Conclusion

Through the combination of two innovative state-of-the-art statistical models, our data suggest a U-shaped association of glucose and mortality in the ICU setting, especially in non-diabetic patients with sepsis, with an optimal average glucose between 160 and 190 mg/dL. These results are in line with current guidelines recommending that intensivists should aim for a liberal glucose range in most patients. Employing target trial emulations in combination with causal inference in high-resolution healthcare data can provide meaningful insights into the relationship between treatment effects and patient outcomes. Even though the causal inference framework in an ICU setting should be viewed as suggestive rather than definitive, the adoption of target trial emulations before planning prospective studies should become the norm as this methodology can help plan costly RCTs.

## Supplementary material

10.1136/bmjopen-2025-104916 online supplemental file 1

## Data Availability

Data are available in a public, open access repository.
