# Supplementary material for: What is the ideal glucose range for a patient with sepsis in the ICU? A retrospective analysis of MIMIC-IV
Source: BMJ Open. 2026 Jan 28;16(1):e104916. doi: 10.1136/bmjopen-2025-104916 (PMC12853541; doi:10.1136/bmjopen-2025-104916)

**What is the ideal glucose range for a patient with sepsis in the ICU? A retrospective analysis of MIMIC-IV**

**RUNNING TITLE: ICU glucose optimum**

**Author List:**

Tristan Struja, MD, MSc, MPH, [tstruja@mit.edu](mailto:tstruja@mit.edu) – 2, 4*

Lasse Hyldig Hansen, MSc, [lassehyldigh@gmail.com](mailto:lassehyldigh@gmail.com) – 2, 7*

João Matos, MSc, [jcmatos@mit.edu](mailto:jcmatos@mit.edu) – 2, 5

Josep Gómez Álvarez, PhD, [josep.goal@gmail.com](mailto:josep.goal@gmail.com) – 8

Alex Pardo, MSc, [alexpardo.5@gmail.com](mailto:alexpardo.5@gmail.com) – 9

Ismini Lourentzou, PhD, [lourent2@illinois.edu](mailto:lourent2@illinois.edu) – 10

Nima S Hejazi, PhD, ORCID: 0000-0002-7127-2789, [nhejazi@hsph.harvard.edu](mailto:nhejazi@hsph.harvard.edu) – 3

Leo Anthony Celi, MD, MS, MPH, ORCID: 0000-0001-6712-6626, [lceli@mit.edu](mailto:lceli@mit.edu) – 1, 2, 3^†^

Andre Kurepa Waschka, PhD, [awaschka@elon.edu](mailto:awaschka@elon.edu) – 6^†^

* Equally contributing first authors

^†^ Equally contributing last authors

**Author Affiliations:**

1. Division of Pulmonary, Critical Care and Sleep Medicine, Beth Israel Deaconess Medical Center, Boston, MA, USA
2. Laboratory for Computational Physiology, Institute for Medical Engineering and Science, Massachusetts Institute of Technology, Cambridge, MA, USA
3. Department of Biostatistics, Harvard T.H. Chan School of Public Health, Boston, MA, USA
4. Medical University Clinic, Kantonsspital Aarau, Aarau, Switzerland
5. Faculty of Engineering of University of Porto, Porto, Portugal
6. Mercer University, Macon, GA, and Elon University, Elon NC, USA
7. Aarhus University, Jens Chr. Skou 2, 8000 Aarhus, Denmark
8. University Hospital of Tarragona Joan XXIII, Rovira Virgili University, Tarragona, Catalonia, Spain.
9. Rovira Virgili University, Tarragona, Catalonia, Spain.
10. School of Information Sciences, University of Illinois at Urbana - Champaign

**Corresponding Author:**

Tristan Struja, MD, MSc, MPH, ORCID 0000-0003-0199-0184

Email: tstruja@mit.edu

Laboratory for Computational Physiology, Massachusetts Institute of Technology, Cambridge, MA, USA

**Conflicts of Interest:**

None of the authors have any conflicts of interest relevant to this work.

**Supplementary material:**

5 tables, 4 figures

## **SUPPLEMENTARY MATERIALS**

### **Supplementary Table 1.** Definition of considered covariates

| **Covariates** | **Description** | **Handling of missing values** |
| --- | --- | --- |
| Time weighted averages | TWA= (time_1_*var_1_+time_2_*var_2_+…+time_n_*var_n_) / total time | Last observation carried forward |
| Glucose | MIMIC-IV Item IDs 220621, 228388, 25664, 226537 | Last observation carried forward |
| Glucose missingness | Indicator equaling 1 in case value was carried forward from last observation | N/A |
| Sex | As provided by dataset | N/A |
| Race Ethnicity | Grouped into Asian, Black, White, Hispanic, and Other as provided by dataset | N/A |
| Insulin | MIMIC-IV Item IDs 229299, 229619, 223257, 223258, 223259, 223260, 223261, 223262  If multiple insulins were given during the same hour, insulin dose was summed up assuming the following weight according to the perceived duration of action: ½ short acting intravenous (2h), ½ short acting subcutaneous (2h), ⅛ medium acting subcutaneous (8h), and 1/24 long acting subcutaneous (24h) | Assume no insulin received if no entries. |
| Carbohydrates | Both enteral and parenteral forms of carbohydrates were considered. Enteral formulations are only stored in mL, as such we assume that 1mL = 0.2g of carbohydrates. Parenteral formulations were converted from concentrations to grams as provided by the dataset, if not provided we assumed 20% carbohydrate content for total parenteral nutritional solutions and 10% for peripheral parenteral nutritional solutions | Assume no carbohydrates received if no entries. |
| Glucocorticoids | Binarization of non-topical formulations of dexamethasone, hydrocortisone, methylprednisolone, and prednisone only | Assume no glucocorticoid received if no entries. |
| BMI | Weight / (Height/100)^2^ as provided by dataset | N/A |
| Charlson comorbidity index | As provided by dataset | N/A |
| Diabetes types | Type 2 diabetes: ICD-10 codes E11.X  Type 1 diabetes: ICD-10 codes E08.X-E10.X and E13.X | No diabetes in case of no ICD-10 code |
| Age | At admission | N/A |
| Elective admission | As provided by dataset, true or false if admission is elective | N/A |
| English proficiency | As provided by dataset, English or limited | N/A |
| Year group | As provided by dataset, three-yearly bins | N/A |
| SOFA | SOFA score with each of its subcomponents: on admission and for the selected 24 hours, aggregated by the maximum value | Assumption of best possible value in case of missing |
| Invasive mechanical ventilation | Ventilation status of "InvasiveVent" or “Trach” | N/A |
| Renal replacement therapy | As provided by dataset, excluding peritoneal dialysis | N/A |
| Vasopressors | As provided by dataset. Vasopressors considered were vasopressin, (nor-) epinephrine, phenylephrine | N/A |
| Major surgery | As provided by item OASIS score from dataset | N/A |
| Pneumonia | ICD-10 codes J09.X, J1X.X, J85.X, J86.X | N/A |
| Nosocomial infection | Combined endpoint of ICD-10 codes T80.211 (central line associated bloodstream infection), T83.511 (catheter associated urinary tract infection), T81.4X (surgical site infection), J95.851 (ventilator associated pneumonia) | N/A |

**Supplementary Table 2.** Joint models’ specifications with a random intercept for initial hour and SOFA score at admission, and random slope per patient for each model

| **Outcome** | **Description** | **Misc.** |
| --- | --- | --- |
| hourly SOFA  (mean SOFA before centering 7.59) | ns(log glucose_i_) + imputed glucose indicator + glucose group + sex + White ethnicity + glucocorticoids + ns(hour_i_) + ns(log carbohydrates_i_) + ns(log insulin_i_) + ns(centered BMI) + ns(centered Charlson index) + diabetes + ns(centered age) + elective admission + English proficient + study year + IMV + RRT + vasopressor + major surgery + pneumonia | control = lmeControl(opt = 'optim')  method = REML  ns = model with restricted cubic spline with 5 knots |
| hourly insulin  (mean insulin before taking natural log 3.37 U) | ns(log glucose_i_) + imputed glucose indicator + glucose group + sex + White ethnicity + glucocorticoids + ns(hour_i_) + ns(log carbohydrates_i_) + ns(centered BMI) + ns(centered Charlson index) + diabetes + ns(centered age) + elective admission + English proficient + study year + IMV + RRT + vasopressor + major surgery + pneumonia | control = lmeControl(opt = 'optim')  method = REML  ns = model with restricted cubic spline with 5 knots |
| mortality  hypoglycemia <80mg/dl  hypoglycemia <50mg/dl  (time to first event) | ns(time averaged log glucose) + glucose group + sex + White ethnicity + glucocorticoids + ns(time averaged log insulin) + ns(time averaged log carbohydrates) + ns(centered BMI) + ns(centered Charlson index) + ns(centered SOFA at admission) + ns(centered age) + diabetes + elective admission + English proficient + study year +IMV + RRT + vasopressor + major surgery + pneumonia | robust standard errors  ns = model with restricted cubic spline with 5 knots |
| joint model | h(t) = h_0_(t) * exp(𝛾*(time to event outcome) + ff(⍺*(hourly SOFA) + β*(hourly insulin))) | ff = functional form for outcome SOFA = area under the curve over the last 24 hours  ff = functional form for outcome insulin = slope over the last 24 hours |

**Supplementary Table 3.** Additional baseline information on the study cohort stratified by diabetes. Diabetes was determined by the presence of billing codes.

|  | **Diabetes absent (N = 3,418)** | **Diabetes present (N = 4,584)** | **Overall (N = 8,002)** |
| --- | --- | --- | --- |
| **Total carbohydrates (grams)**  **Median (IQR)** | 128 (59.2, 393) | 128 (55.0, 343) | 128 (57.2, 368) |
| **Missing** | 24 (0.7%) | 97 (2.1%) | 121 (1.5%) |
| **Total Proteins (grams)**  **Median (IQR)** | 62.8 (18.8, 148) | 56.6 (19.1, 133) | 59.3 (19.0, 139) |
| **Missing** | 2,422 (70.9%) | 3,288 (71.7%) | 5,710 (71.4%) |
| **Total Calories (kcal)**  **Median (IQR)** | 546 (256, 1,570) | 459 (196, 1,330) | 498 (222, 1,430) |
| **Missing** | 28 (0.8%) | 107 (2.3%) | 135 (1.7%) |
| **Time-weighted average carbohydrates (grams)**  **Mean (SD)** | 1,120 (4,690) | 1,570 (8,460) | 1,380 (7,110) |
| **Time-weighted average methylprednisolone equivalent* (mg)**  **Mean (SD)** | 3.77 (30.7) | 3.18 (21.7) | 3.43 (25.9) |
| **Full Code upon admission** | 3,295 (96.4%) | 4,334 (94.5%) | 7,629 (95.3%) |
| **Full Code upon discharge** | 3,183 (93.1%) | 4,204 (91.7%) | 7,387 (92.3%) |
| **Diabetes with complications** | N/A | 1,585 (34.6%) | 1,585 (19.8%) |
| **Diabetes types** |  |  |  |
| Type 1 Diabetes | N/A | 498 (10.9%) | 498 (6.2%) |
| Type 2 Diabetes | N/A | 4,086 (89.1%) | 4,086 (51.1%) |
| **Hypertension** | 2,214 (64.8%) | 3,734 (81.5%) | 5,948 (74.3%) |
| **Heart failure** | 1,075 (31.5%) | 2,189 (47.8%) | 3,264 (40.8%) |
| **Asthma** | 39 (1.1%) | 64 (1.4%) | 103 (1.3%) |
| **COPD** | 746 (21.8%) | 1,243 (27.1%) | 1,989 (24.9%) |
| **Chronic kidney disease** | 271 (7.9%) | 986 (21.5%) | 1,257 (15.7%) |

**Abbreviations:** SD, standard deviation; IQR; interquartile range; BMI, body mass index; COPD, chronic obstructive pulmonary disease; t-w, time-weighted; N/A, not applicable;

**Legend:** *Information was retrieved from the chart and dose was averaged over the time given.

**Supplementary Table 4.** Joint modeling full results of the longitudinal models only

| **Joint model (beta coefficient (95% CR))** | | | |
| --- | --- | --- | --- |
| **Variable \| Outcome** | **Mortality** | **Hypoglycemia mild**  **< 80 mg/dl** | **Hypoglycemia severe**  **< 50 mg/dl** |
| **Longitudinal model - Outcome hourly SOFA** | | | |
| **Intercept** | -1.7 (-1.9 to -1.5) | -1.7 (-1.9 to -1.5) | -1.7 (-1.9 to -1.5) |
| **log glucose spline 1** | 0.3 (0.2 to 0.3) | 0.3 (0.2 to 0.4) | 0.3 (0.2 to 0.4) |
| **log glucose spline 2** | 0.3 (0.2 to 0.3) | 0.3 (0.2 to 0.4) | 0.3 (0.2 to 0.4) |
| **log glucose spline 3** | 0.0 (0.0 to 0.1) | 0.1 (0.0 to 0.1) | 0.1 (0.0 to 0.1) |
| **log glucose spline 4** | 0.4 (0.2 to 0.6) | 0.4 (0.3 to 0.6) | 0.4 (0.3 to 0.6) |
| **log glucose spline 5** | -0.3 (-0.4 to -0.2) | -0.3 (-0.4 to -0.2) | -0.3 (-0.4 to -0.2) |
| **Glucose LOCF indicator** | -0.0 (-0.0 to 0.0) | -0.0 (-0.0 to 0.0) | -0.0 (-0.0 to 0.0) |
| **TW glucose group 141-180 mg/dl** | Reference | | |
| **TW glucose group < 141 mg/dl** | 0.0 (-0.0 to 0.0) | 0.0 (-0.0 to 0.0) | 0.0 (-0.0 to 0.0) |
| **TW glucose group > 180 mg/dl** | 0.0 (0.0 to 0.1) | 0.0 (0.0 to 0.1) | 0.0 (0.0 to 0.1) |
| **Female sex** | -0.1 (-0.1 to -0.1) | -0.1 (-0.1 to -0.1) | -0.1 (-0.1 to -0.1) |
| **White ethnicity** | 0.0 (-0.0 to 0.0) | 0.0 (-0.0 to 0.0) | 0.0 (-0.0 to 0.0) |
| **hour of admission spline 1** | -0.1 (-0.1 to -0.1) | -0.1 (-0.1 to -0.1) | -0.1 (-0.1 to -0.1) |
| **hour of admission spline 2** | -0.7 (-0.8 to -0.7) | -0.7 (-0.8 to -0.7) | -0.7 (-0.8 to -0.7) |
| **hour of admission spline 3** | -7.7 (-7.9 to -7.6) | -7.8 (-7.9 to -7.6) | -7.7 (-7.9 to -7.6) |
| **hour of admission spline 4** | -14.2 (-14.5 to -14.0) | -14.3 (-14.5 to -14.0) | -14.2 (-14.5 to -13.9) |
| **hour of admission spline 5** | -21.3 (-21.7 to -21.0) | -21.5 (-21.8 to -21.1) | -21.3 (-21.8 to -20.9) |
| **log carbohydrates spline 1** | -0.0 (-0.1 to 0.1) | -0.0 (-0.1 to 0.1) | -0.0 (-0.1 to 0.1) |
| **log carbohydrates spline 2** | -0.0 (-0.1 to 0.1) | -0.0 (-0.1 to 0.1) | -0.0 (-0.1 to 0.1) |
| **log carbohydrates spline 3** | -0.1 (-0.1 to -0.1) | -0.1 (-0.1 to -0.0) | -0.1 (-0.1 to -0.1) |
| **log carbohydrates spline 4** | -0.2 (-0.4 to -0.1) | -0.2 (-0.4 to -0.0) | -0.2 (-0.4 to -0.1) |
| **log carbohydrates spline 5** | -0.0 (-0.1 to -0.0) | -0.0 (-0.1 to -0.0) | -0.0 (-0.1 to -0.0) |
| **Glucocorticoids** | -0.0 (-0.1 to 0.0) | -0.0 (-0.1 to 0.0) | -0.0 (-0.1 to 0.0) |
| **log insulin spline 1** | 0.2 (0.2 to 0.2) | 0.2 (0.2 to 0.2) | 0.2 (0.2 to 0.2) |
| **log insulin spline 2** | 0.2 (0.2 to 0.2) | 0.2 (0.2 to 0.2) | 0.2 (0.2 to 0.2) |
| **log insulin spline 3** | 0.2 (0.1 to 0.2) | 0.2 (0.1 to 0.2) | 0.2 (0.1 to 0.2) |
| **log insulin spline 4** | 0.5 (0.5 to 0.5) | 0.5 (0.5 to 0.5) | 0.5 (0.5 to 0.5) |
| **log insulin spline 5** | 0.1 (0.1 to 0.1) | 0.1 (0.1 to 0.1) | 0.1 (0.1 to 0.1) |
| **BMI spline 1** | 0.0 (-0.1 to 0.1) | 0.0 (-0.1 to 0.1) | 0.0 (-0.1 to 0.1) |
| **BMI spline 2** | 0.1 (-0.0 to 0.2) | 0.1 (0.0 to 0.2) | 0.1 (-0.0 to 0.2) |
| **BMI spline 3** | 0.1 (0.0 to 0.2) | 0.1 (0.0 to 0.2) | 0.1 (0.0 to 0.2) |
| **BMI spline 4** | 0.1 (-0.1 to 0.4) | 0.1 (-0.1 to 0.4) | 0.1 (-0.1 to 0.3) |
| **BMI spline 5** | -0.0 (-0.2 to 0.1) | -0.0 (-0.2 to 0.1) | -0.0 (-0.2 to 0.1) |
| **Charlson index spline 1** | 0.5 (0.4 to 0.6) | 0.5 (0.4 to 0.6) | 0.5 (0.4 to 0.6) |
| **Charlson index spline 2** | 0.6 (0.5 to 0.7) | 0.6 (0.5 to 0.7) | 0.6 (0.5 to 0.7) |
| **Charlson index spline 3** | 0.6 (0.5 to 0.8) | 0.6 (0.5 to 0.8) | 0.6 (0.5 to 0.7) |
| **Charlson index spline 4** | 0.8 (0.6 to 1.1) | 0.8 (0.6 to 1.1) | 0.9 (0.6 to 1.1) |
| **Charlson index spline 5** | 0.3 (0.0 to 0.6) | 0.3 (-0.0 to 0.6) | 0.3 (0.0 to 0.6) |
| **Diabetes mellitus** | -0.2 (-0.2 to -0.1) | -0.2 (-0.2 to -0.1) | -0.2 (-0.2 to -0.1) |
| **Age spline 1** | -0.2 (-0.3 to -0.0) | -0.2 (-0.3 to -0.0) | -0.2 (-0.3 to -0.0) |
| **Age spline 2** | -0.2 (-0.3 to -0.0) | -0.2 (-0.3 to -0.0) | -0.2 (-0.3 to -0.0) |
| **Age spline 3** | -0.2 (-0.3 to -0.1) | -0.2 (-0.3 to -0.1) | -0.2 (-0.3 to -0.1) |
| **Age spline 4** | -0.1 (-0.4 to 0.2) | -0.1 (-0.4 to 0.2) | -0.1 (-0.4 to 0.2) |
| **Age spline 5** | -0.2 (-0.3 to -0.1) | -0.2 (-0.3 to -0.1) | -0.2 (-0.3 to -0.1) |
| **Elective admission** | -0.1 (-0.1 to -0.0) | -0.1 (-0.1 to -0.0) | -0.1 (-0.1 to -0.0) |
| **English proficient** | -0.0 (-0.1 to 0.0) | -0.0 (-0.1 to 0.0) | -0.0 (-0.1 to 0.0) |
| **Year 2008-2010** | Reference | | |
| **Year 2011-2013** | 0.0 (-0.0 to 0.0) | 0.0 (-0.0 to 0.0) | 0.0 (-0.0 to 0.0) |
| **Year 2014-2016** | 0.0 (-0.0 to 0.1) | 0.0 (-0.0 to 0.1) | 0.0 (-0.0 to 0.1) |
| **Year 2017-2019** | 0.1 (0.1 to 0.1) | 0.1 (0.1 to 0.1) | 0.1 (0.1 to 0.1) |
| **IMV** | 0.3 (0.2 to 0.3) | 0.3 (0.2 to 0.3) | 0.3 (0.2 to 0.3) |
| **RRT** | 0.8 (0.7 to 0.8) | 0.8 (0.7 to 0.8) | 0.8 (0.7 to 0.8) |
| **Vasopressor** | 0.4 (0.4 to 0.5) | 0.4 (0.4 to 0.5) | 0.4 (0.4 to 0.5) |
| **Major surgery** | -0.1 (-0.2 to -0.1) | -0.1 (-0.2 to -0.1) | -0.1 (-0.2 to -0.1) |
| **Pneumonia** | -0.1 (-0.2 to -0.0) | -0.1 (-0.2 to -0.0) | -0.1 (-0.2 to -0.0) |
| **Longitudinal model - Outcome hourly insulin** | | | |
| **Intercept** | -2.9 (-3.3 to -2.6) | -2.8 (-3.1 to -2.5) | -2.9 (-3.2 to -2.5) |
| **log glucose spline 1** | 0.4 (0.3 to 0.6) | 0.4 (0.2 to 0.5) | 0.3 (0.1 to 0.5) |
| **log glucose spline 2** | 0.4 (0.3 to 0.6) | 0.4 (0.2 to 0.5) | 0.4 (0.2 to 0.5) |
| **log glucose spline 3** | 0.8 (0.7 to 0.9) | 0.8 (0.7 to 0.9) | 0.8 (0.7 to 0.9) |
| **log glucose spline 4** | 2.0 (1.7 to 2.4) | 1.9 (1.6 to 2.2) | 1.9 (1.5 to 2.2) |
| **log glucose spline 5** | 0.8 (0.7 to 1.0) | 0.8 (0.7 to 1.0) | 0.8 (0.6 to 1.0) |
| **Glucose LOCF indicator** | -0.1 (-0.1 to -0.1) | -0.1 (-0.1 to -0.1) | -0.1 (-0.1 to -0.1) |
| **TW glucose group 141-180 mg/dl** | Reference | | |
| **TW glucose group < 141 mg/dl** | -0.0 (-0.0 to 0.0) | -0.0 (-0.1 to 0.0) | -0.0 (-0.0 to 0.0) |
| **TW glucose group > 180 mg/dl** | 0.2 (0.2 to 0.2) | 0.2 (0.2 to 0.2) | 0.2 (0.2 to 0.2) |
| **Female sex** | -0.1 (-0.1 to -0.0) | -0.1 (-0.1 to -0.0) | -0.1 (-0.1 to -0.0) |
| **White ethnicity** | -0.0 (-0.1 to 0.0) | -0.0 (-0.1 to 0.0) | -0.0 (-0.1 to 0.0) |
| **log carbohydrates spline 1** | -0.4 (-0.5 to -0.2) | -0.4 (-0.5 to -0.2) | -0.4 (-0.5 to -0.2) |
| **log carbohydrates spline 2** | -0.4 (-0.5 to -0.2) | -0.4 (-0.6 to -0.2) | -0.4 (-0.5 to -0.2) |
| **log carbohydrates spline 3** | 0.0 (-0.1 to 0.1) | 0.0 (-0.1 to 0.1) | 0.0 (-0.1 to 0.1) |
| **log carbohydrates spline 4** | -0.8 (-1.1 to -0.4) | -0.9 (-1.2 to -0.5) | -0.8 (-1.2 to -0.5) |
| **log carbohydrates spline 5** | -0.1 (-0.2 to -0.0) | -0.1 (-0.2 to -0.0) | -0.1 (-0.2 to -0.0) |
| **Glucocorticoids** | -0.1 (-0.1 to -0.0) | -0.1 (-0.1 to -0.0) | -0.1 (-0.1 to -0.0) |
| **BMI spline 1** | 2.3 (2.3 to 2.3) | 2.3 (2.3 to 2.3) | 2.3 (2.3 to 2.3) |
| **BMI spline 2** | 2.6 (2.6 to 2.7) | 2.6 (2.6 to 2.7) | 2.6 (2.6 to 2.7) |
| **BMI spline 3** | -3.1 (-3.4 to -2.9) | -3.1 (-3.4 to -2.6) | -3.2 (-3.5 to -2.8) |
| **BMI spline 4** | -1.8 (-2.2 to -1.2) | -1.7 (-2.4 to -0.8) | -1.9 (-2.4 to -1.1) |
| **BMI spline 5** | -9.8 (-10.5 to -9.0) | -9.7 (-10.7 to -8.5) | -10.0 (-10.7 to -8.9) |
| **hour of admission spline 1** | 0.2 (0.1 to 0.4) | 0.2 (0.1 to 0.4) | 0.2 (0.1 to 0.4) |
| **hour of admission spline 2** | 0.3 (0.1 to 0.4) | 0.3 (0.1 to 0.4) | 0.3 (0.1 to 0.4) |
| **hour of admission spline 3** | 0.4 (0.3 to 0.5) | 0.4 (0.3 to 0.5) | 0.4 (0.3 to 0.5) |
| **hour of admission spline 4** | 0.5 (0.3 to 0.8) | 0.5 (0.3 to 0.8) | 0.5 (0.3 to 0.8) |
| **hour of admission spline 5** | 0.3 (0.1 to 0.4) | 0.3 (0.1 to 0.5) | 0.3 (0.1 to 0.4) |
| **Charlson index spline 1** | 0.2 (0.0 to 0.3) | 0.2 (0.0 to 0.3) | 0.2 (0.0 to 0.3) |
| **Charlson index spline 2** | 0.2 (0.1 to 0.4) | 0.2 (0.1 to 0.4) | 0.2 (0.1 to 0.4) |
| **Charlson index spline 3** | 0.0 (-0.1 to 0.2) | 0.0 (-0.1 to 0.2) | 0.0 (-0.1 to 0.2) |
| **Charlson index spline 4** | 0.1 (-0.2 to 0.5) | 0.1 (-0.2 to 0.5) | 0.1 (-0.2 to 0.5) |
| **Charlson index spline 5** | -0.1 (-0.5 to 0.3) | -0.1 (-0.5 to 0.3) | -0.1 (-0.5 to 0.3) |
| **Diabetes mellitus** | 0.1 (0.1 to 0.2) | 0.1 (0.1 to 0.2) | 0.1 (0.1 to 0.2) |
| **Age spline 1** | -0.2 (-0.4 to -0.0) | -0.2 (-0.4 to -0.0) | -0.2 (-0.4 to -0.0) |
| **Age spline 2** | -0.2 (-0.4 to 0.0) | -0.2 (-0.4 to 0.0) | -0.2 (-0.4 to 0.0) |
| **Age spline 3** | -0.2 (-0.3 to -0.0) | -0.2 (-0.3 to -0.0) | -0.2 (-0.3 to -0.0) |
| **Age spline 4** | -0.3 (-0.6 to 0.1) | -0.3 (-0.6 to 0.1) | -0.3 (-0.7 to 0.1) |
| **Age spline 5** | -0.1 (-0.3 to -0.0) | -0.1 (-0.3 to -0.0) | -0.1 (-0.3 to -0.0) |
| **Elective admission** | 0.1 (0.0 to 0.1) | 0.1 (0.0 to 0.1) | 0.1 (0.0 to 0.1) |
| **English proficient** | -0.0 (-0.1 to 0.0) | -0.0 (-0.1 to 0.0) | -0.0 (-0.1 to 0.0) |
| **Year 2008-2010** | Reference | | |
| **Year 2011-2013** | 0.0 (-0.0 to 0.0) | 0.0 (-0.0 to 0.1) | 0.0 (-0.0 to 0.1) |
| **Year 2014-2016** | 0.1 (0.0 to 0.1) | 0.1 (0.0 to 0.1) | 0.1 (0.0 to 0.1) |
| **Year 2017-2019** | -0.0 (-0.1 to 0.0) | -0.0 (-0.1 to 0.0) | -0.0 (-0.1 to 0.0) |
| **IMV** | 0.0 (0.0 to 0.1) | 0.0 (0.0 to 0.1) | 0.0 (0.0 to 0.1) |
| **RRT** | 0.1 (0.0 to 0.1) | 0.1 (0.0 to 0.1) | 0.1 (0.0 to 0.1) |
| **Vasopressor** | 0.1 (0.1 to 0.1) | 0.1 (0.1 to 0.1) | 0.1 (0.1 to 0.1) |
| **Major surgery** | 0.3 (0.3 to 0.3) | 0.3 (0.3 to 0.3) | 0.3 (0.3 to 0.3) |
| **Pneumonia** | 0.0 (-0.1 to 0.1) | 0.0 (-0.1 to 0.1) | 0.0 (-0.1 to 0.1) |

**Abbreviations**: CI, confidence interval; CR, Credible Interval; TW, time weighted averaged; LOCF, last observation carried forward; BMI, body mass index; SOFA, sequential organ failure assessment score; IMV, invasive mechanical ventilation; RRT, renal replacement therapy
**Legend**: In the longitudinal models, each subject was allowed to have a random slope, and a random intercept per hour of observation and admission SOFA value.

**Supplementary Table 5.** Joint modeling results of survival models only

| **Joint model (hazard ratio (95% CR))** | | | |
| --- | --- | --- | --- |
| **Variable \| Outcome** | **Mortality** | **Hypoglycemia mild**  **< 80 mg/dl** | **Hypoglycemia severe**  **< 50 mg/dl** |
| **Survival model - Outcome time to event** | | | |
| **log TW glucose spline 1** | 0.1 (0.0 to 0.4) | 0.0 (0.0 to 0.1) | 0.0 (0.0 to 0.0) |
| **log TW glucose spline 2** | 0.1 (0.0 to 0.5) | 0.0 (0.0 to 0.1) | 0.0 (0.0 to 0.0) |
| **log TW glucose spline 3** | 0.4 (0.2 to 1.1) | 0.0 (0.0 to 0.1) | 0.0 (0.0 to 0.4) |
| **log TW glucose spline 4** | 0.0 (0.0 to 0.6) | 0.0 (0.0 to 0.0) | 0.0 (0.0 to 0.0) |
| **log TW glucose spline 5** | 16.1 (4.6 to 54.4) | 0.0 (0.0 to 0.0) | 0.0 (0.0 to 0.0) |
| **TW glucose group 141-180 mg/dl** | Reference | | |
| **TW glucose group < 141 mg/dl** | 0.9 (0.6 to 1.3) | 0.9 (0.8 to 1.1) | 0.6 (0.3 to 1.0) |
| **TW glucose group > 180 mg/dl** | 1.2 (0.9 to 1.7) | 1.1 (0.8 to 1.4) | 0.8 (0.4 to 1.5) |
| **Female sex** | 1.1 (1.0 to 1.3) | 1.1 (1.1 to 1.2) | 1.4 (1.2 to 1.7) |
| **White ethnicity** | 1.2 (1.1 to 1.4) | 1.1 (1.0 to 1.2) | 1.1 (0.9 to 1.3) |
| **log TW insulin spline 1** | 0.5 (0.1 to 2.6) | 1.1 (0.5 to 3.0) | 1.8 (0.4 to 14.5) |
| **log TW insulin spline 2** | 0.4 (0.1 to 2.1) | 1.5 (0.6 to 4.1) | 2.0 (0.4 to 15.8) |
| **log TW insulin spline 3** | 0.7 (0.3 to 1.8) | 6.5 (3.5 to 12.5) | 10.8 (3.0 to 41.9) |
| **log TW insulin spline 4** | 0.9 (0.1 to 27.4) | 0.6 (0.1 to 4.7) | 4.0 (0.1 to 292.6) |
| **log TW insulin spline 5** | 0.3 (0.0 to 2.0) | 1.6 (0.4 to 4.9) | 4.0 (0.3 to 32.2) |
| **log TW carbohydrates spline 1** | 1.4 (0.7 to 3.0) | 1.3 (0.8 to 2.1) | 5.1 (0.6 to 69.9) |
| **log TW carbohydrates spline 2** | 1.0 (0.5 to 2.3) | 0.9 (0.6 to 1.4) | 7.2 (0.9 to 103.0) |
| **log TW carbohydrates spline 3** | 0.6 (0.3 to 1.3) | 0.6 (0.4 to 0.9) | 23.8 (4.2 to 152.1) |
| **log TW carbohydrates spline 4** | 1.6 (0.3 to 9.9) | 0.9 (0.3 to 2.4) | 5.2 (0.1 to 1605.8) |
| **log TW carbohydrates spline 5** | 0.7 (0.2 to 2.5) | 0.9 (0.4 to 2.0) | 5.1 (0.4 to 57.6) |
| **Glucocorticoids** | 0.9 (0.7 to 1.1) | 0.9 (0.7 to 1.0) | 0.8 (0.5 to 1.1) |
| **BMI spline 1** | 0.6 (0.4 to 0.9) | 0.8 (0.6 to 1.0) | 0.4 (0.2 to 0.7) |
| **BMI spline 2** | 0.6 (0.4 to 0.9) | 0.8 (0.5 to 1.0) | 0.3 (0.2 to 0.6) |
| **BMI spline 3** | 0.5 (0.3 to 0.7) | 0.4 (0.3 to 0.6) | 0.1 (0.1 to 0.3) |
| **BMI spline 4** | 0.4 (0.2 to 1.0) | 0.5 (0.3 to 1.1) | 0.1 (0.0 to 0.5) |
| **BMI spline 5** | 0.7 (0.3 to 1.2) | 0.3 (0.2 to 0.6) | 0.2 (0.1 to 0.7) |
| **Charlson index spline 1** | 0.6 (0.4 to 1.1) | 1.6 (1.2 to 2.2) | 2.1 (0.8 to 5.4) |
| **Charlson index spline 2** | 0.6 (0.3 to 1.0) | 1.5 (1.1 to 2.1) | 1.9 (0.7 to 5.6) |
| **Charlson index spline 3** | 1.7 (1.1 to 2.9) | 1.8 (1.2 to 2.6) | 2.5 (0.9 to 7.0) |
| **Charlson index spline 4** | 0.7 (0.2 to 2.5) | 2.7 (1.2 to 6.1) | 1.8 (0.1 to 22.3) |
| **Charlson index spline 5** | 4.7 (2.0 to 10.6) | 1.5 (0.6 to 3.8) | 0.3 (0.0 to 4.2) |
| **Diabetes mellitus** | 0.7 (0.6 to 0.8) | 1.8 (1.6 to 2.0) | 3.4 (2.6 to 4.4) |
| **Age spline 1** | 1.3 (0.7 to 2.7) | 0.9 (0.7 to 1.3) | 0.8 (0.4 to 1.9) |
| **Age spline 2** | 1.4 (0.7 to 2.9) | 0.7 (0.5 to 1.0) | 0.9 (0.3 to 2.4) |
| **Age spline 3** | 1.9 (1.2 to 3.1) | 1.0 (0.7 to 1.3) | 1.1 (0.5 to 2.2) |
| **Age spline 4** | 1.9 (0.5 to 8.6) | 0.5 (0.3 to 1.2) | 0.5 (0.1 to 3.5) |
| **Age spline 5** | 2.2 (1.5 to 3.3) | 0.9 (0.7 to 1.1) | 0.4 (0.2 to 0.9) |
| **Elective admission** | 0.4 (0.3 to 0.6) | 1.0 (0.9 to 1.1) | 0.8 (0.6 to 1.2) |
| **English proficient** | 1.5 (1.2 to 1.7) | 1.2 (1.1 to 1.4) | 1.3 (1.0 to 1.8) |
| **Year 2008-2010** | Reference | | |
| **Year 2011-2013** | 1.0 (0.9 to 1.1) | 0.9 (0.8 to 1.0) | 0.7 (0.6 to 0.9) |
| **Year 2014-2016** | 1.1 (0.9 to 1.3) | 0.8 (0.7 to 0.9) | 0.7 (0.5 to 0.9) |
| **Year 2017-2019** | 1.1 (0.9 to 1.2) | 0.8 (0.7 to 0.9) | 0.6 (0.4 to 0.8) |
| **SOFA admission spline 1** | 1.5 (0.9 to 2.4) | 1.9 (1.4 to 2.5) | 2.1 (1.0 to 4.7) |
| **SOFA admission spline 2** | 1.2 (0.7 to 2.2) | 1.8 (1.3 to 2.6) | 1.4 (0.6 to 3.6) |
| **SOFA admission spline 3** | 1.1 (0.7 to 1.6) | 1.9 (1.4 to 2.6) | 2.3 (1.1 to 5.0) |
| **SOFA admission spline 4** | 1.6 (0.5 to 5.4) | 7.2 (3.4 to 15.3) | 2.7 (0.4 to 21.1) |
| **SOFA admission spline 5** | 1.0 (0.5 to 2.0) | 13.4 (6.9 to 25.5) | 2.2 (0.5 to 9.6) |
| **IMV** | 0.8 (0.7 to 0.9) | 0.6 (0.5 to 0.7) | 0.4 (0.3 to 0.5) |
| **RRT** | 0.6 (0.5 to 0.6) | 1.1 (1.0 to 1.3) | 1.1 (0.9 to 1.5) |
| **Vasopressor** | 0.9 (0.8 to 1.1) | 1.0 (0.9 to 1.1) | 0.9 (0.7 to 1.2) |
| **Major surgery** | 0.6 (0.5 to 0.7) | 0.7 (0.7 to 0.8) | 0.7 (0.5 to 0.8) |
| **Pneumonia** | 0.9 (0.7 to 1.2) | 0.9 (0.7 to 1.1) | 0.9 (0.5 to 1.5) |
| **Association hourly SOFA**  **(area last 24h)** | 2.3 (2.1 to 2.4) | 0.5 (0.4 to 0.5) | 0.8 (0.7 to 1.0) |
| **Association hourly insulin**  **(slope last 24h)** | 0.0 (0.0 to 0.0) | 0.0 (0.0 to 0.0) | 0.0 (0.0 to 0.0) |

**Abbreviations**: CI, confidence interval; CR, Credible Interval; TW, time weighted averaged; BMI, body mass index; SOFA, sequential organ failure assessment score; IMV, invasive mechanical ventilation; RRT, renal replacement therapy

**Legend**: In the longitudinal models, each subject was allowed to have a random slope, and a random intercept per hour of observation and admission SOFA value.

### **Supplementary Figure 1.** TMLE derived average treatment effects over the five strata of mean glucose, no stratification by diabetes
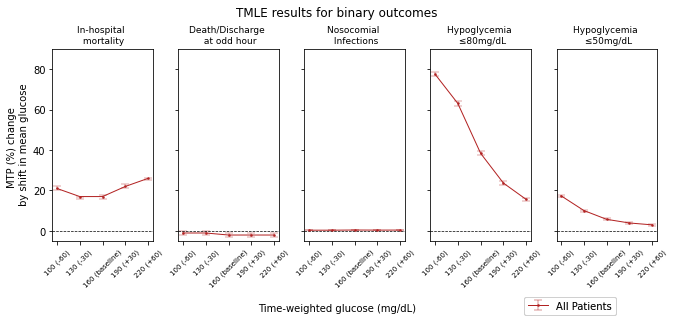
status; Panel A Binary outcomes; Panel B Continuous outcomes. All patients included.

**A**


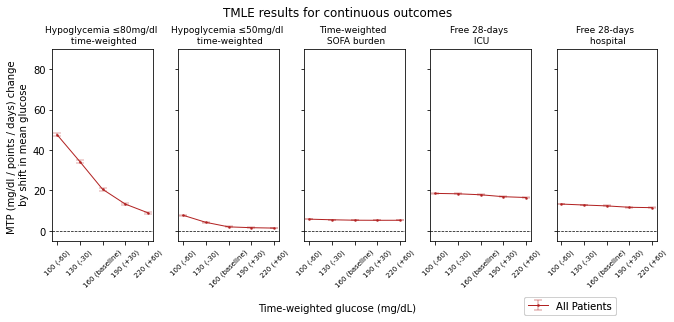
**B**

###

**Abbreviations:** MTP, modified counterfactual treatment-policy risk; TMLE, targeted maximum likelihood

### **Supplementary Figure 2.** Shifts in glucose distributions used in TMLE models. All patients included. TMLE framework shifts the entire distribution by a set value from the mean without changing the distribution’s characteristics.

### **
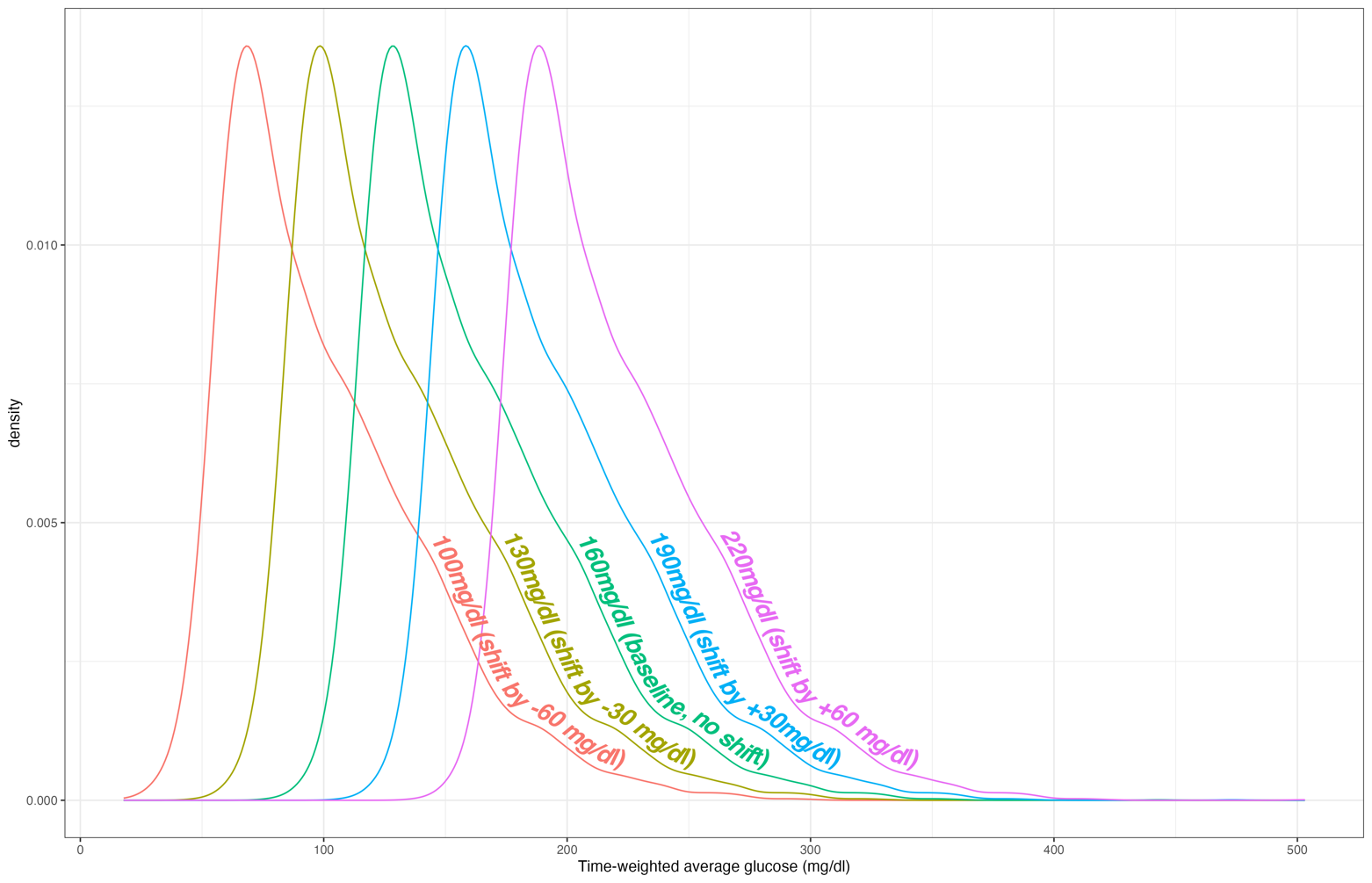
**

###

### **Supplementary Figure 3.** Negative control outcome for the survival model - discharge or death at even versus odd hour


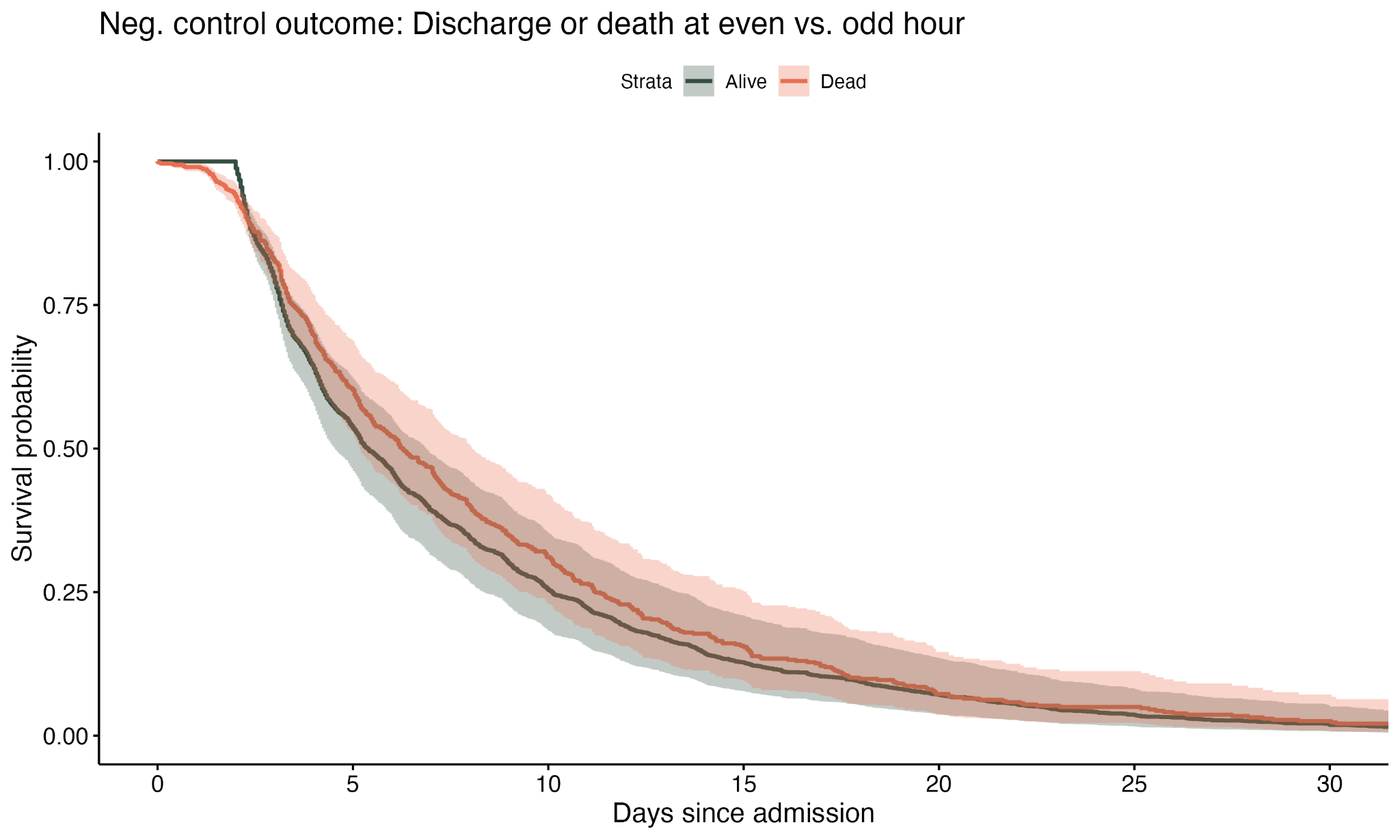


**Supplementary Figure 4.** Cox model derived survival curves split by time-weighted average glucose into bins matching current guidelines’ suggestions for both patients with and without diabetes.


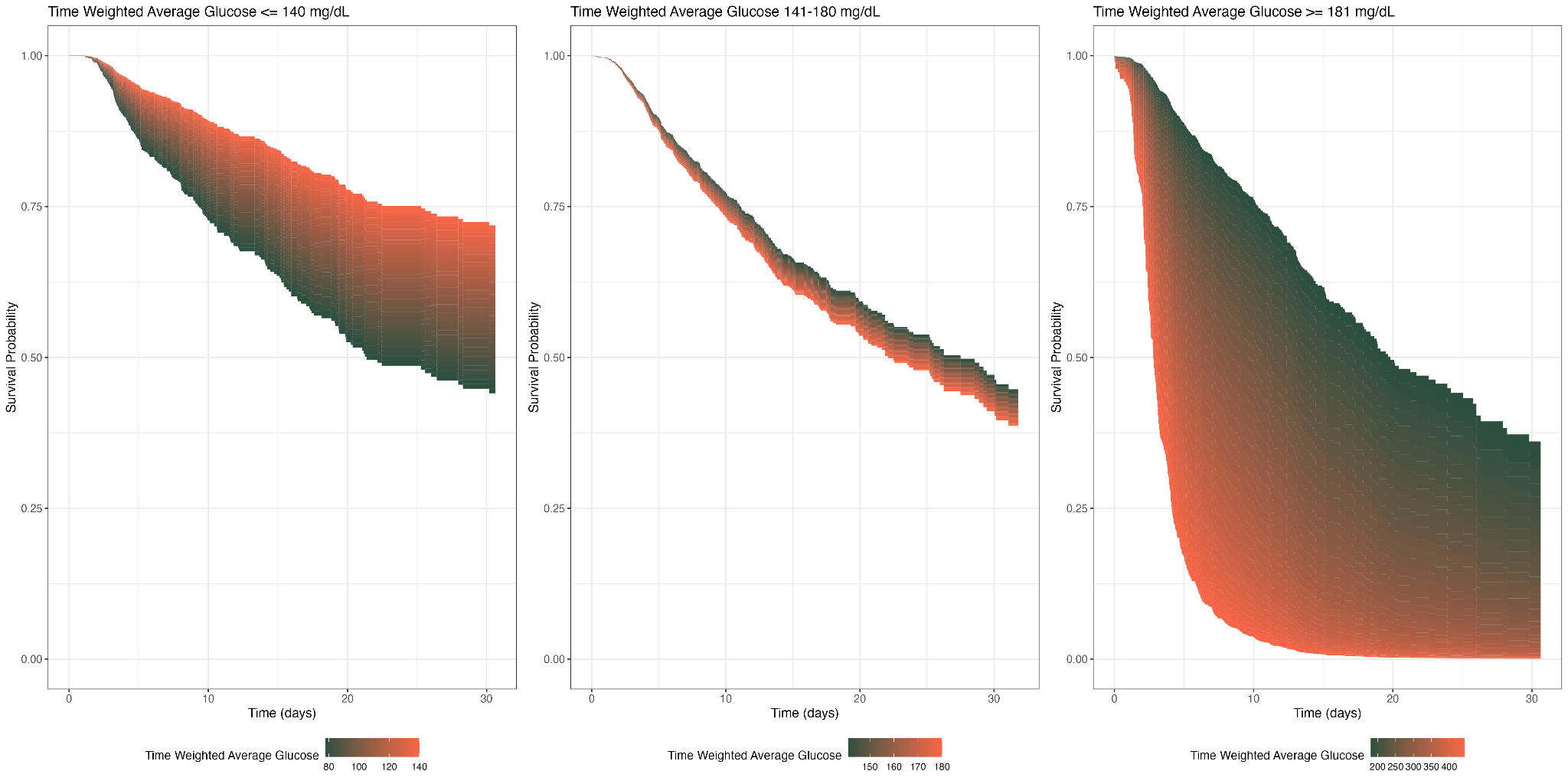


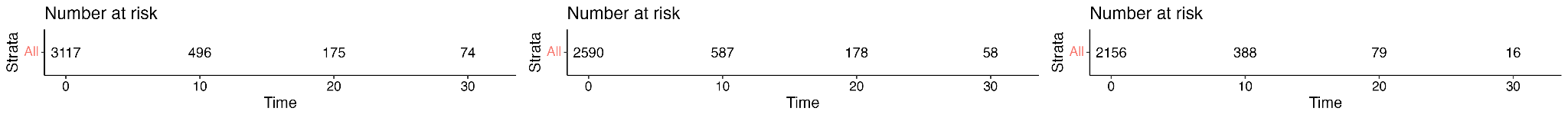

Supplement: online supplemental file 1 [file bmjopen-16-1-s001.docx]
