# Supplementary material for: What is the ideal glucose range for a patient with sepsis in the ICU? A retrospective analysis of MIMIC-IV
Source: BMJ Open. 2026 Jan 28;16(1):e104916. doi: 10.1136/bmjopen-2025-104916 (PMC12853541; doi:10.1136/bmjopen-2025-104916)
Supplement: Supplementary file 2 [file bmjopen-2025-104916.reviewer_comments.pdf]

PEER REVIEW HISTORY

BMJ Open publishes all reviews undertaken for accepted manuscripts. Reviewers are asked to complete a checklist review form and are provided with free text boxes to elaborate on their assessment. These free text comments are reproduced below.

ARTICLE DETAILS

Title (Provisional)

What is the ideal glucose range for a patient with sepsis in the ICU? A retrospective analysis of MIMIC-IV

Authors

Struja, Tristan; Hansen, Lasse; Matos, Joao; Gómez, Josep; Pardo, Alex; Lourentzou, Ismini; Hejazi, Nima; Celi, Leo; Waschka, Andre

---

VERSION 1 - REVIEW

**Reviewer**	**1**
**Name**	**Kelson, Zoe**
**Affiliation**	**University of Exeter, Mathematics**
**Date**	**19-Jun-2025**
**COI**	**None**

This study aims to investigate the optimal glucose ranges in ICU patients with sepsis independent of confounding covariates.

Reviewer comments:

"While there was no association between glucose ranges and mortality in patients with diabetes, there was a U-shaped curve in nondiabetic patients with a mortality of 17% when glucose range at 130mg/dl (IQR 55-322) and mortality rates of 20-26% when glucose was lower or higher. Hypoglycemic events (<40mg/dl) were significantly more frequent in the lowest glucose range of 100mg/dl (IQR 25-294)."

Can the authors please report statistical outcomes alongside study findings in the Abstract?

"This study is reported in accordance with the Strengthening the Reporting of Observational studies in Epidemiology (STROBE) statement"

Thanks for providing a copy of the STROBE checklist.

"MIMIC-IV includes physiologic data collected from bedside monitors, as well as other clinical variables and provider documents. "

and

"Patient-level variables were obtained from the database at the time of ICU admission and timevarying variables were aggregated by calculating the time-weighted average for the TMLE and survival analyses (e.g. Sequential Organ Failure Assessment (SOFA) score burden was calculated for every hour in the ICU; in case of missing values the last observation was carried forward; and an hourly weighted average for the whole stay was calculated – see supplementary table 1). For joint modeling, we extracted time-varying variables at hourly intervals of glucose measurements (see supplementary table 1). We used closest-neighbor matching for other timevarying variables."

Can the authors please comment on whether missing data can be assumed to be missing at random?

Can the authors please consider undertaking a sensitivity analysis on the approach taken for missing data?

"Targeted trial emulation of glucose ranges using causal inference targeted maximum likelihood estimation and longitudinal mixed-effects models combined with survival models" [Abstract]

and

"We used TMLE models (tmle3 package, version 0.2) adjusted for confounders to compute the Modified counterfactual treatment-policy risks (MTP, or more loosely the shifted average treatment effect) for distinct shifts from the mean time-weighted glucose average 15. For counterfactual modeling, we used the SuperLearner3 package, an ensemble machine learning algorithm. Five-fold crossvalidation was performed for each model. "

and

"we also pursued a joint modeling approach using the R package JMBayes2 (version 0.4.5) 27. This package utilizes Markov chain Monte Carlo (MCMC) algorithms for fitting joint models. In our study, we simultaneously fitted two longitudinal linear mixed effects models, with the SOFA score and insulin dosages as outcomes, and hourly glucose levels alongside other confounders as predictors. We allowed for a random slope for each subject and random intercept per hour and admission SOFA value. "

and

"used to estimate the evolution towards discharge or death in a Cox proportional hazards model."

and

"All continuous predictors were mean centered and fitted with natural cubic splines with 5 knots (see supplementary Table 2 for model specifications). Bayesian models were fitted over 5 chains with 16,000 iterations each (burn-in phase 4,000) and non-informative priors"

A suitable approach and appropriate modelling methods have been applied by the authors.

"Single-center, academic referral hospital in Boston, Massachusetts, USA... Of 73,181 eligible patients, 8,002 patients with a median age of 66 years (41% women, 67% White, 57% diabetic patients) were included" [Abstract]

and

"After selecting our cohort, we had a sample size of 8,002 admissions (see Figure 1). Table 1 depicts the baseline characteristics of our cohort stratified by diabetes status (for additional data see supplementary Table 3)."

Whilst the authors acknowledge that this is a single center study in the discussion of the limitations, can they please further comment on whether they consider the cohort to be demographically and characteristically representative?

"Using a causal inference framework to assess critically ill patients with sepsis..."

Can the authors please further discuss the conditions for inferring causality in this study (e.g. is it assumed that all confounders are known and adjusted for)?

"The main limitation of our analysis is that we only have data from one academic center in the United States. Resources and protocols can vary across healthcare providers and thus add an important layer to the generalizability of every study. However, we are confident that our results are robust, as they are well in line with the current evidence from RCTs. Even though other publicly available ICU databases exist, they either lack the high-resolution in data points of MIMICIV (e.g. eICU-CRD) or do not provide essential diagnoses and demographics albeit having a higher resolution in data points (e.g. AmsterdamUMCdb, HiRID) 31 . Although our sample size was similar to concurrent RCTs, our sample size might be insufficient to detect smaller benefits, especially given the narrowing support of data on both tails of the distribution. But even if such benefits exist, they need to be weighed against the clear and steep increase in risk for hypoglycemia."

A discussion on the study limitations is provided by the authors.

**Reviewer**	**2**
**Name**	**Qiu, Haibo**
**Affiliation**	**Southeast University Zhongda Hospital**
**Date**	**13-Nov-2025**
**COI**	**None**

Summary

Thanks for giving this chance to review this paper. This paper examined glucose management in ICU sepsis patients using the MIMIC-IV database, employing Targeted Maximum Likelihood Estimation (TMLE) and joint modeling to assess various glucose target ranges. The findings suggested that while tight glucose control does not improve clinical

outcomes, it increased the risk of hypoglycemia. Lower glucose ranges were associated with more ICU- and hospital-free days but did not reduce mortality. While the study provides valuable insights into glucose control for ICU sepsis patients, there are several limitations that must be addressed.

Major points

1. The research objective of this paper seems unclear. The title asks, What is the ideal glucose range for a patient with sepsis in the ICU? but the end of introduction section mentioned “the questions on which patient is benefitting the most”. The conclusion only discussed the U-shaped association between the glucose and mortality without specifying the ideal range. Please provide clarification.

2. Please explain why the five glucose ranges used in the TMLE analysis were pre-defined. How were their interquartile ranges derived? Why not directly determine the optimal glucose control range through non-linear methods (e.g., Loess curves or segmented logistic regression)?

Minor points

1. Please explain why TMLE was chosen for analysis. Other methods like nonlinear mixed-effects models and generalized additive models, could also be used as sensitivity analyses to enhance the robustness of conclusions. Additionally, since authors aimed to identify subgroups, unsupervised clustering might be more appropriate than categorizing patients into diabetes and non-diabetes subgroups.

2. Please explain why different missing data handling methods were used for different time-varying variables. Forward imputation was used for SOFA, while closest-neighbor matching was applied for other variables. Both methods may introduce bias.

3. The Statistical Analysis section in the methodology is not clear and should be better restructured.

4. The data collection period is not mentioned in the paper. Please add it.

5. For the Results, Table 1 usually summarizes the data according to the primary outcome (in-hospital mortality in this paper).

6. Apart from time-weighted average glucose, glucose is a highly variable value. You may consider evaluating other variables like glycemic coefficient of variation.

7. The study found a higher risk of hypoglycemia in patients with tighter glucose control. However, it did not address in detail how insulin dosing was managed or how adjustments might have contributed to hypoglycemic events.

8. The conclusion in the abstract does not align well with the conclusion in the main body of the article. Please check it.

9. The Discussion section should include an explanation for the negative results obtained in the study but simplify the discussion on the relationship between parenteral nutrition and changes in glucose or outcomes.

VERSION 1 - AUTHOR RESPONSE

Reviewer: 1

Prof. Zoe Kelson, University of Exeter
Comments to the Author:
This study aims to investigate the optimal glucose ranges in ICU patients with sepsis independent of confounding covariates.

Reviewer comments:

"While there was no association between glucose ranges and mortality in patients with diabetes, there was a U-shaped curve in nondiabetic patients with a mortality of 17% when glucose range at 130mg/dl (IQR 55-322) and mortality rates of 20-26% when glucose was lower or higher. Hypoglycemic events (<40mg/dl) were significantly more frequent in the lowest glucose range of 100mg/dl (IQR 25-294)."
Can the authors please report statistical outcomes alongside study findings in the Abstract?
Reply: Thank you for pointing out our negligent inconsistencies. We have also implemented suggestions by the other reviewer and rewritten the second half of the abstract and hope it is to your liking now.
The abstract now reads as follows:

"Importance: Clinical trials have produced inconclusive results regarding the optimal glucose range for a patient with sepsis in the intensive care unit (ICU) receiving insulin treatment.
Objective: To investigate the optimal glucose range in ICU-patients with sepsis independent of confounding covariates.
Design: Targeted trial emulation of glucose ranges using causal inference targeted maximum likelihood estimation and longitudinal mixed-effects models combined with survival models.
Setting: Single-center, academic referral hospital in Boston, Massachusetts, USA.
Participants: Adults fulfilling sepsis-3 criteria with at least three glucose readings and insulin treatment from the MIMIC-IV database (2008-2019).
Exposure: Five predefined glucose distributions with means at 100mg/dl, 130mg/dl, 160mg/dl (baseline), 190mg/dl, and 220mg/dl mimicking current guidelines' recommendations (140-180 mg/dl).
Main outcome and measure: The primary outcome was in-hospital mortality. Modified counterfactual treatment-policy-risks (MTP) across distinct time-weighted glucose ranges were estimated.
Results: Of 73,181 eligible patients, 8,002 patients with a median age of 66 years (41% women, 67% White, 57% diabetes) were included. There was a U-shaped curve between glucose range and mortality in patients without diabetes, but overall, this association was not significant (mean glucose at 100mg/dl with 21% mortality and mean glucose at 220mg/dl with 26% mortality, p-for-trend 0.26). Mortality was lowest at 17% with mean glucose between 130-160mg/dl. Hypoglycemic events (<80mg/dl) became increasingly more frequent with tighter

glucose control 16% at 220mg/dl compared to 77% at 100mg/dl (p-for-trend 0.01). Joint modeling corroborated these results and did not identify covariates that would favor lower glucose ranges in subsets of patients.
Conclusion and Relevance: Our data suggests a U-shaped association of glucose and mortality with an optimal average glucose between 160-190mg/dl. These results confirm current guideline recommendations. Together with recent results from randomized controlled trials, intensivists should aim for a liberal glucose range in most patients."

"This study is reported in accordance with the Strengthening the Reporting of Observational studies in Epidemiology (STROBE) statement"
Thanks for providing a copy of the STROBE checklist.
Reply: Thank you for your kind words.

"MIMIC-IV includes physiologic data collected from bedside monitors, as well as other clinical variables and provider documents. "
and
"Patient-level variables were obtained from the database at the time of ICU admission and timevarying variables were aggregated by calculating the time-weighted average for the TMLE and survival analyses (e.g. Sequential Organ Failure Assessment (SOFA) score burden was calculated for every hour in the ICU; in case of missing values the last observation was carried forward; and an hourly weighted average for the whole stay was calculated – see supplementary table 1). For joint modeling, we extracted time-varying variables at hourly intervals of glucose measurements (see supplementary table 1). We used closest-neighbor matching for other timevarying variables."
Can the authors please comment on whether missing data can be assumed to be missing at random?
Reply: Thank you for this important point. All clinical databases have variable patterns of missingness. This is even more pronounced in large observational ICU databases such as MIMIC-IV (Johnson, A.E.W. et al. MIMIC-IV, a freely accessible electronic health record dataset. Sci Data ***10****, 1 (2023). https://doi.org/10.1038/s41597-022-01899-x). Patterns of missingness vary by variable type, clinical workflow, ICU location, and most importantly clinical judgement. For physiologic variables derived from bedside monitors, missingness is usually driven by clinical workflow (e.g., temporary sensor disconnection, device downtime) rather than by patient prognosis, and some studies suggest that these data can often be treated as missing at random (MAR).*
However, laboratory values, medications, and documentations, usually exhibit missing not at random (MNAR), because sicker patients tend to be measured more frequently in an effort to better monitor and treat their disease course to finally improve prognosis.
This has been demonstrated by our senior author Leo Celi in two publications looking at glucose and insulin administration in MIMIC data (Baker L et al. Real-world characterization of blood glucose control and insulin use in the intensive care unit. Sci Rep. 2020 Jul 1;10(1):10718. doi: 10.1038/s41598-020-67864-z and Robles Arévalo A, et al. Data-driven curation process for describing the blood glucose management in the intensive care unit. Sci Data. 2021 Mar 10;8(1):80. doi: 10.1038/s41597-021-00864-4). Therein, the authors corroborated that glucose measurement and insulin administration are tightly related to patient illness and preceding events such as hypoglycemia.

In a general matter, these observations are also well known for two other publicly available ICU databases, eICU-CRD (O'Halloran HM, et al. Characterizing the Patients, Hospitals, and Data Quality of the eICU Collaborative Research Database. Crit Care Med. 2020 Dec;48(12):1737-1743. doi: 10.1097/CCM.0000000000004633) and the Amsterdam UMCdb intensive care dataset (Williams CYK et al, Application of the Sepsis-3 criteria to describe sepsis epidemiology in the Amsterdam UMCdb intensive care dataset. PLoS One. 2024 Jun 21;19(6):e0304133. doi: 10.1371/journal.pone.0304133).

We have addressed these questions in two separate works by looking at the occurrence of missingness of lactate and glucose (Pradhan P, et al. Potential source of bias in AI models: lactate measurement in the ICU in sepsis patients as a template. Front Med (Lausanne). 2025 Jul 9;12:1606254. doi: 10.3389/fmed.2025.1606254, and Teotia K, Jia Y, Link Woite N, Celi LA, Matos J, Struja T. Variation in monitoring: Glucose measurement in the ICU as a case study to preempt spurious correlations. J Biomed Inform. 2024 May;153:104643. doi: 10.1016/j.jbi.2024.104643). Lactate is also a very frequently measured blood value which has good prognostic properties in sepsis.
In these two projects, we found that there were no differences in lactate measurements between patients according to ethnic background. Regarding glucose measurements there were very slight differences with males (incidence rate ratio (IRR) 1.06, 95% confidence interval (CI) 1.01 - 1.21), patients who identify themselves as Hispanic (IRR 1.11, 95% CI 1.01 - 1.21), or Black (IRR 1.06, 95% CI 1.01 - 1.12), and patients being English proficient (IRR 1.08, 95% CI 1.01 - 1.15) having had higher chances of subsequent glucose readings.

Given this evidence, we assumed missingness to be approximately MNAR for most variables, but are confident that these slight variations did not bias our results as our findings are also well in line with data from RCTs. However, we acknowledged this limitation by adding the following sentences to the limitations section, please see below at your follow-up question.

Can the authors please consider undertaking a sensitivity analysis on the approach taken for missing data?
Reply: Thank you very much for this thoughtful suggestion. We fully agree that, in principle, sensitivity analyses for missing data can be valuable. However, in our specific setting we believe that an additional missing-data sensitivity analysis would not provide new insights for the following reasons:

1. ***Restricting the analysis to patients with complete data is barely possible.*** *Glucose measurements and insulin administration are highly variable on a patient's disease course. As such, there is will be no patient with a complete dataset, and analysis would have to be restricted on an arbitrary cut-off, which itself is highly likely of introducing bias.*
2. ***Most imputation methods assume data are missing at random (MAR).*** *As discussed in our previous response, this is not a reasonable assumption for many time-varying clinical variables, particularly laboratory values and interventions. Applying imputation approaches in a context where missingness is predominantly MNAR would therefore risk introducing additional bias rather than reducing it.*
3. ***We already conducted a conceptual sensitivity analysis by using two fundamentally different statistical frameworks - TMLE and joint modeling.*** *These two approaches rely on distinct assumptions about the data-generating process and treat time-varying information very differently. The fact that both produced consistent*

results which also are in line with evidence from RCTs increases our confidence that missingness patterns did not meaningfully alter our conclusions.

4. ***Within the joint model, we additionally incorporated a glucose-LOCF indicator variable** (see Supplementary Table 5). This allowed us to test whether the presence of imputed glucose values influenced the longitudinal or survival components of the model. This predictor was not associated with outcomes (beta coefficients and 95% CRs for mortality -0.0 (-0.0 to 0.0), for mild hypoglycemia -0.0 (-0.0 to 0.0), and for severe hypoglycemia -0.0 (-0.0 to 0.0), suggesting that the LOCF procedure did not introduce detectable bias.*

Given these considerations, and the extensive analyses already performed, we believe that an additional formal sensitivity analysis would be unlikely to yield interpretable or clinically meaningful findings. Nevertheless, we have strengthened our limitations section to explicitly acknowledge the challenges of MNAR missingness and its potential impact:

“Time-varying covariates had missing values handled via last observation carried forward or assumed absence, which may introduce bias if missingness was not random. Missingness in large observational ICU databases such as MIMIC-IV is complex and often driven by clinical workflow, severity of illness, and provider judgment rather than random processes, as demonstrated in prior work. For physiologic monitor–derived variables, missingness may approximate missingness at random, but for laboratory measurements, medications, and interventions, accumulating evidence indicates predominantly missingness not at random patterns. Our own recent analyses of lactate and glucose monitoring using MIMIC-IV data further support that measurement frequency reflects clinical acuity rather than demographic factors alone. Because standard imputation methods generally rely on missingness at random assumptions, alternative imputation strategies may introduce additional bias rather than mitigate it. Additionally, previous studies including the recent SOFA-2 score development and a Massachusetts-based ICU analysis suggest that alternative imputation methods do not substantially alter results in such settings. Reassuringly, we incorporated an imputation indicator for glucose measurement in the joint model and used two distinct analytic frameworks that yielded concordant results, but naturally residual bias from missing data mechanisms can never be fully excluded.”

"Targeted trial emulation of glucose ranges using causal inference targeted maximum likelihood estimation and longitudinal mixed-effects models combined with survival models" [Abstract]
and
"We used TMLE models (tmle3 package, version 0.2) adjusted for confounders to compute the Modified counterfactual treatment-policy risks (MTP, or more loosely the shifted average treatment effect) for distinct shifts from the mean time-weighted glucose average 15. For counterfactual modeling, we used the SuperLearner3 package, an ensemble machine learning algorithm. Five-fold crossvalidation was performed for each model. "
and
"we also pursued a joint modeling approach using the R package JMBayes2 (version 0.4.5) 27. This package utilizes Markov chain Monte Carlo (MCMC) algorithms for fitting joint models. In our study, we simultaneously fitted two longitudinal linear mixed effects models, with the SOFA score and insulin dosages as outcomes, and hourly glucose levels alongside other

confounders as predictors. We allowed for a random slope for each subject and random intercept per hour and admission SOFA value. "
and
"used to estimate the evolution towards discharge or death in a Cox proportional hazards model."
and
"All continuous predictors were mean centered and fitted with natural cubic splines with 5 knots (see supplementary Table 2 for model specifications). Bayesian models were fitted over 5 chains with 16,000 iterations each (burn-in phase 4,000) and non-informative priors"
A suitable approach and appropriate modelling methods have been applied by the authors.
Reply: Thank you for your positive feedback. We want to highlight that we have made semantical updates to the cited section because of suggestions provided by the other reviewer.

"Single-center, academic referral hospital in Boston, Massachusetts, USA... Of 73,181 eligible patients, 8,002 patients with a median age of 66 years (41% women, 67% White, 57% diabetic patients) were included" [Abstract]
and
"After selecting our cohort, we had a sample size of 8,002 admissions (see Figure 1). Table 1 depicts the baseline characteristics of our cohort stratified by diabetes status (for additional data see supplementary Table 3)."
Whilst the authors acknowledge that this is a single center study in the discussion of the limitations, can they please further comment on whether they consider the cohort to be demographically and characteristically representative?
Reply: Thank you for this important point. We agree that the fact our data come from a single academic referral center warrants careful consideration regarding representativeness.
According to the U.S. Census Bureau, the state of Massachusetts has a population of roughly 7 million (2024 estimate, https://data.census.gov/profile/Massachusetts?g=040XX00US25).
In that population, as of 2024 roughly 69% identify as White, ~7% as Black/African American, ~12% as Hispanic, and ~7% as Asian alone. The median age of Massachusetts is about 40 years. The Massachusetts Department of Public Health (https://www.mass.gov/orgs/population-health-information-tool) estimated the diabetes prevalence about 9.3% of adults in 2016.

Because our cohort is older, more comorbid, and drawn from a hospital referral center, it cannot be considered representative of the general Massachusetts (or U.S.) adult population. Instead, it represents a high-risk, hospitalized subpopulation. The very similar distribution of race-ethnicity compared to state-level census suggests that our cohort should be very generalizable. The very high diabetes fraction of 57% confirms that the cohort is highly selected, and thus any conclusions should be interpreted as applying to a hospitalized, comorbid population, not to the broader community.

We added the following paragraph to the limitations section:
“We note that our cohort differs substantially from the general population of Massachusetts. State census data indicate a median age of ~40 years and a prevalence of diagnosed diabetes around 9% among adults, compared with a median age of 66 years and 57% diabetes rate in our sample. On the other hand, race-ethnicity of cohort was very similar to census data were roughly 69% of Massachusetts’ residents identify as White. As such, our cohort likely

represents a medically complex, older and more comorbid population than community-based samples, which limits generalizability."

"Using a causal inference framework to assess critically ill patients with sepsis..."
Can the authors please further discuss the conditions for inferring causality in this study (e.g. is it assumed that all confounders are known and adjusted for)?
Reply: We thank the reviewer for this important question. Our analysis draws conceptually on the counterfactual causal inference framework articulated by Hernán and Robins (Hernán & Robins, Causal Inference: What If, Chapman & Hall/CRC, 2020). Within this framework, valid causal inference from observational data requires several key assumptions:

1) Exchangeability (No unmeasured confounding):
Conditional on the measured covariates, the potential outcomes are independent of treatment assignment. This means that all confounders that jointly influence both exposure and outcome must be measured and appropriately adjusted for. As with most observational studies in critical care, we cannot guarantee that all confounders are known or fully captured in the dataset. We therefore explicitly acknowledge that residual confounding remains possible.

2) Positivity:
Each patient must have a non-zero probability of receiving each exposure level, conditional on their covariates. We examined the covariate distributions and did not identify structural violations of positivity (see Table 1 bottom). However, as Hernán and Robins emphasize, violations can still occur in highly selected clinical cohorts limiting generalizability.

3) Consistency:
The treatment under study must be well-defined, such that each individual's observed outcome corresponds to their counterfactual outcome for that exposure. We aimed to ensure clear and reproducible definitions for exposures and outcomes following the principles recommended by Hernán and Robins.

4) Correct model specification:
Causal effect estimates rely on adequate model specification for adjustment. Consistent with best practices described in Causal Inference: What If, we modeled covariates based on clinical knowledge and prior literature, but acknowledge that model misspecification may still contribute to bias.

We therefore rephrased our results as causally suggestive rather than definitive, and we explicitly discuss in the manuscript that our conclusions rest on these assumptions. Given the complexity of sepsis patients and the limitations inherent to retrospective data, unmeasured confounding cannot be fully excluded.

Accordingly, we rephrased the discussion section as follows:
Valid interpretation of our estimates relies on several core assumptions of the causal inference framework as outlined by Hernán and Robins [29]. These include exchangeability (i.e., absence of unmeasured confounding after adjustment), positivity (i.e., non-zero probability of receiving each treatment level within covariate strata), consistency (i.e., well-defined and comparable exposure interventions), and adequate model specification. Although TMLE mitigates model

misspecification through data-adaptive estimation, we acknowledge that the assumption of no unmeasured confounding can hardly be ever verified in any observational ICU research. Consequently, our findings should be interpreted as causally suggestive rather than definitive.

"The main limitation of our analysis is that we only have data from one academic center in the United States. Resources and protocols can vary across healthcare providers and thus add an important layer to the generalizability of every study. However, we are confident that our results are robust, as they are well in line with the current evidence from RCTs. Even though other publicly available ICU databases exist, they either lack the high-resolution in data points of MIMICIV (e.g. eICU-CRD) or do not provide essential diagnoses and demographics albeit having a higher resolution in data points (e.g. AmsterdamUMCdb, HiRID). Although our sample size was similar to concurrent RCTs, our sample size might be insufficient to detect smaller benefits, especially given the narrowing support of data on both tails of the distribution. But even if such benefits exist, they need to be weighed against the clear and steep increase in risk for hypoglycemia."
A discussion on the study limitations is provided by the authors.
Reply: Thank you for your kind words and willingness to review our work .

Reviewer: 2
Dr. Haibo Qiu, Southeast University Zhongda Hospital
Comments to the Author:

Summary
Thanks for giving this chance to review this paper. This paper examined glucose management in ICU sepsis patients using the MIMIC-IV database, employing Targeted Maximum Likelihood Estimation (TMLE) and joint modeling to assess various glucose target ranges. The findings suggested that while tight glucose control does not improve clinical outcomes, it increased the risk of hypoglycemia. Lower glucose ranges were associated with more ICU- and hospital-free days but did not reduce mortality. While the study provides valuable insights into glucose control for ICU sepsis patients, there are several limitations that must be addressed.
Reply: Thank you for valuable feedback.

Major points
1. The research objective of this paper seems unclear. The title asks, What is the ideal glucose range for a patient with sepsis in the ICU? but the end of introduction section mentioned “the questions on which patient is benefitting the most”. The conclusion only discussed the U-shaped association between the glucose and mortality without specifying the ideal range. Please provide clarification.
Reply: Thank you very much for pointing out these inconsistencies. We have now changed the abstract, introduction and discussion to all align with our initially posed question in our title. We further implemented suggestions from question 1 by the other reviewer.

The sections now read as follows:

Abstract:
Importance: Clinical trials have produced inconclusive results regarding the optimal glucose range for a patient with sepsis in the intensive care unit (ICU) receiving insulin treatment.

Objective: To investigate the optimal glucose range in ICU-patients with sepsis independent of confounding covariates.
Design: Targeted trial emulation of glucose ranges using causal inference targeted maximum likelihood estimation and longitudinal mixed-effects models combined with survival models.
Setting: Single-center, academic referral hospital in Boston, Massachusetts, USA.
Participants: Adults fulfilling sepsis-3 criteria with at least three glucose readings and insulin treatment from the MIMIC-IV database (2008-2019).
Exposure: Five predefined glucose distributions with means at 100mg/dl, 130mg/dl, 160mg/dl (baseline), 190mg/dl, and 220mg/dl mimicking current guidelines' recommendations (140-180 mg/dl).
Main outcome and measure: The primary outcome was in-hospital mortality. Modified counterfactual treatment-policy-risks (MTP) across distinct time-weighted glucose ranges were estimated.
Results: Of 73,181 eligible patients, 8,002 patients with a median age of 66 years (41% women, 67% White, 57% diabetes) were included. There was a U-shaped curve between glucose range and mortality in patients without diabetes, but overall, this association was not significant (mean glucose at 100mg/dl with 21% mortality and mean glucose at 220mg/dl with 26% mortality, p-for-trend 0.26). Mortality was lowest at 17% with mean glucose between 130-160mg/dl. Hypoglycemic events (<80mg/dl) became increasingly more frequent with tighter glucose control 16% at 220mg/dl compared to 77% at 100mg/dl (p-for-trend 0.01). Joint modeling corroborated these results and did not identify covariates that would favor lower glucose ranges in subsets of patients.
Conclusion and Relevance: Our data suggests a U-shaped association of glucose and mortality with an optimal average glucose between 160-190mg/dl. These results confirm current guideline recommendations. Together with recent results from randomized controlled trials, intensivists should aim for a liberal glucose range in most patients."

Introduction:
"…With this combination, finding an optimal glucose range for patients with sepsis using real-world data from high-resolution datasets such as MIMIC-IV becomes possible; a task which could be too costly, impractical, or even unethical to for an RCT. We hypothesized that baseline covariates might predict which patients with sepsis in the ICU might benefit from lower glucose ranges."

Discussion:
"Using a causal inference framework in a large cohort of critically ill patients with sepsis, we found no evidence that tighter glucose control with higher insulin exposure improved any clinically meaningful outcome. Across all TMLE analyses, glucose-lowering was not associated with reduced mortality, reduced SOFA burden, fewer nosocomial infections, or more hospital- or ICU-free days. Instead, lower glucose ranges consistently increased the risk of hypoglycemia. An average glucose between 160 to 190mg/dl seemed to be the optimal compromise. These findings were corroborated by joint modeling, which integrated hourly glucose values and patient-specific trajectories, but was not able to identify a covariate pattern or subgroup that benefited from lower glucose ranges…"

Conclusion:
"Through the combination of two innovative state-of-the-art statistical models, our data suggests a U-shaped association of glucose and mortality in the ICU setting, especially in non-

diabetic sepsis patients, with an optimal average glucose between 160 to 190mg/dl. These results are in line with current guidelines recommending that intensivists should aim for a liberal glucose range in most patients. Employing target trial emulations in combination with causal inference in high-resolution healthcare data can provide meaningful insights into the relationship between treatment effects and patient outcomes. Even though the casual inference framework in an ICU setting should be viewed at as suggestive rather than definitive, the adoption of target trial emulations before planning prospective studies should become the norm as this methodology can help plan costly RCTs."

2. Please explain why the five glucose ranges used in the TMLE analysis were pre-defined. How were their interquartile ranges derived? Why not directly determine the optimal glucose control range through non-linear methods (e.g., Loess curves or segmented logistic regression)?

Reply: Thank you for this thoughtful comment. While non-linear, data-adaptive methods as proposed are valuable for exploratory visualization, they typically yield ranges that are harder to translate into actionable clinical targets. Given our aim to evaluate clinically recognizable treatment strategies for target trial emulation, we considered predefined and guidelines-aligned categories more appropriate.

We chose the five predefined glucose ranges to align our analysis with the glucose targets used in the recent large randomized trial by Gunst et al. (NEJM 2023) and with current guideline-recommended ranges. Our intention was to enhance clinical interpretability and comparability with existing literature rather than to derive data-driven categories that might be less intuitive to practicing clinicians.

On re-examination, we realized that the interquartile ranges depicted in supplemental Figure 2 were not accurate, and we have corrected this mistake (see below). The TMLE framework shifts the entire distribution by a set value from the mean without changing the distribution's characteristics. As the mean time-weighted average in our cohort was 160mg/dl, we did shift the distribution by -60, -30, 0, +30, and +60mg/dl. We further rephrased the abstract and the methods section.

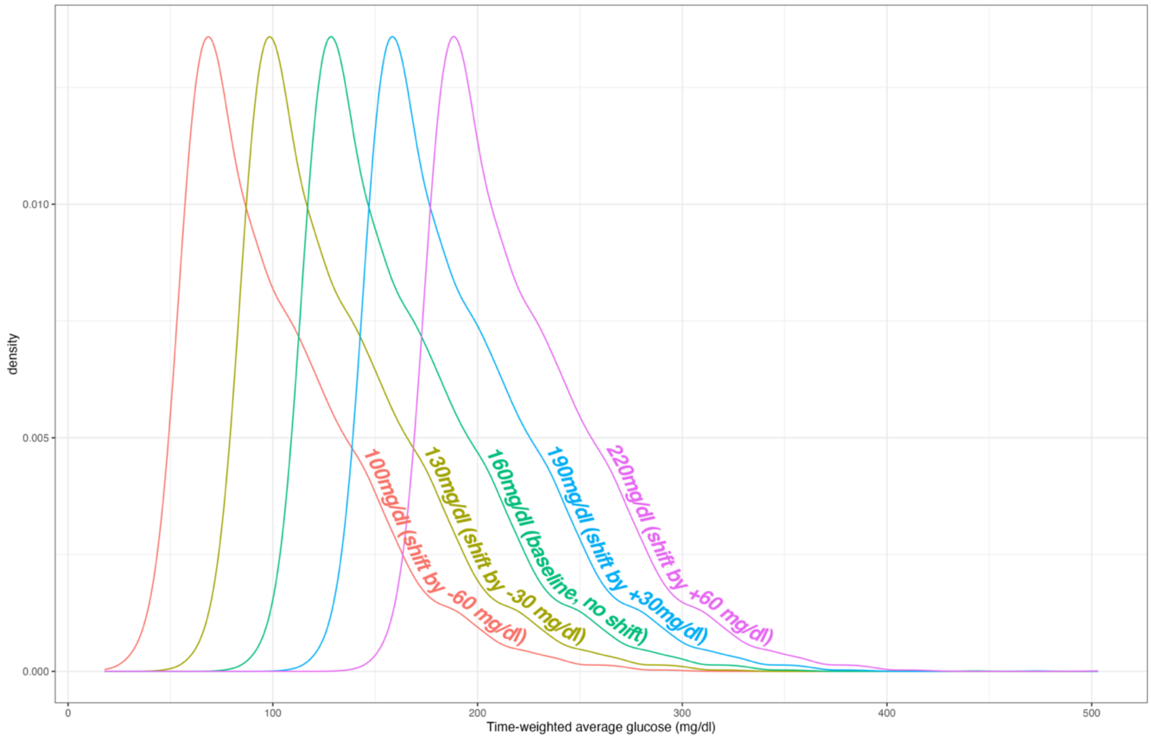

Minor points

1. Please explain why TMLE was chosen for analysis. Other methods like nonlinear mixed-effects models and generalized additive models, could also be used as sensitivity analyses to enhance the robustness of conclusions. Additionally, since authors aimed to identify subgroups, unsupervised clustering might be more appropriate than categorizing patients into diabetes and non-diabetes subgroups.

Reply: We appreciate the reviewer's comments and the opportunity to clarify our analytic choices. TMLE was selected because our primary objective was to obtain causal effect estimates of glycemic exposure on clinical outcomes. TMLE offers several advantages that align well with the structure of our data:

- *Double robustness: valid estimates as long as either the outcome model or the treatment/exposure model is correctly specified.*
- *Machine-learning integration: flexible incorporation of Super Learner algorithms into models (see point above), reducing reliance on parametric assumptions.*
- *Bias reduction under model misspecification: Particularly relevant given the nonlinear trajectories and potential time-varying confounding present in our dataset.*

These properties make TMLE well suited for causal inference in complex clinical datasets which was our main motivation for selecting it.

We agree that nonlinear mixed-effects models and generalized additive models can be informative tools for modelling longitudinal trajectories. However, we would like to clarify that our analysis already includes joint modelling integrating linear mixed-effects models for longitudinal markers together with time-to-event models. Joint modelling addresses the primary issues such as modelling patient-specific trajectories, accounting for correlation between repeated measures, and avoiding bias due to informative dropout by linking longitudinal and survival components that usually motivate mixed-effects or GAM approaches.

Because of this, we do not believe the suggested additional models would provide conceptual advantages beyond what our joint modelling framework already captures. They could be used as sensitivity analyses, but would not address limitations that our models would have not already mitigated.

Thank you also for highlighting the possibility of unsupervised clustering to detect latent subgroups. While clustering is valuable in exploratory phenotyping, our subgroup definitions were clinically prespecified (i.e., diabetes vs. non-diabetes) and based on strong mechanistic and epidemiological rationale. Unsupervised clustering may discover mathematically distinct groups, but their biological or clinical interpretability is uncertain, and not in line with our goal of clinical inference.

Acknowledging your deliberations, we added the following sentence to the discussion section:
"Although mixed-effects or generalized additive models-based approaches could be used descriptively, our joint modelling framework already incorporates the longitudinal correlation structure."

2. Please explain why different missing data handling methods were used for different time-varying variables. Forward imputation was used for SOFA, while closest-neighbor matching was applied for other variables. Both methods may introduce bias.
Reply: We acknowledge that we handled missing values differently. We tried to handle missing values in accordance with the other, similar studies. Forward imputation (i.e., last observation carried forward, LOCF) for SOFA scores, and closest neighbor (i.e., assumed zeros for medications) are common methods in retrospective ICU research. We agree that both methods can introduce bias if missingness is not random. However, current literature suggests that this problem is not of paramount relevance while other imputation methods are much more labor intensive. The authors of the very recent SOFA 2 score utilized multiple imputations methods without detecting substantially different results (doi 10.1001/jama.2025.20516). This was also corroborated in a local analysis (doi 10.1097/ccm.0000000000006338). As such, we do not believe our approach introduced substantial bias, but we acknowledge your concerns and those of the other reviewer (see above) by adding this sentence to the limitations section:

"Time-varying covariates had missing values handled via last observation carried forward or assumed absence, which may introduce bias if missingness was not random. Missingness in large observational ICU databases such as MIMIC-IV is complex and often driven by clinical workflow, severity of illness, and provider judgment rather than random processes, as demonstrated in prior work. For physiologic monitor–derived variables, missingness may approximate missingness at random, but for laboratory measurements, medications, and interventions, accumulating evidence indicates predominantly missingness not at random patterns. Our own recent analyses of lactate and glucose monitoring using MIMIC-IV data further support that measurement frequency reflects clinical acuity rather than demographic factors alone. Because standard imputation methods generally rely on missingness at random assumptions, alternative imputation strategies may introduce additional bias rather than mitigate it. Additionally, previous studies including the recent SOFA-2 score development and a Massachusetts-based ICU analysis suggest that alternative imputation methods do not substantially alter results in such settings. Reassuringly, we incorporated an imputation indicator for glucose measurement in the joint model and used two distinct analytic frameworks

that yielded concordant results, but naturally residual bias from missing data mechanisms can never be fully excluded."

3. The Statistical Analysis section in the methodology is not clear and should be better restructured.
Reply: Thank you for your feedback. We have rephrased the statistical analyses section as follows and hope it is clearer now:

"Statistical analyses were performed using R version 4.3.1 and Python 3.10. We applied two complementary analytical frameworks: (1) targeted maximum likelihood estimation (TMLE) to estimate causal effects of glucose shifts on clinical outcomes, and (2) joint longitudinal–survival modeling to examine associations between glycemic trajectories, organ dysfunction as per SOFA score, and mortality.

For causal estimation, we used TMLE implemented in the tmle3 package (version 0.2). In this framework, the exposure (time-weighted mean glucose) is conceptualized as the treatment variable. We estimated a modified counterfactual treatment-policy risk (MTP, or more loosely the shifted average treatment effect), defined as counterfactual shifts of the observed glucose distribution by prespecified amounts while preserving its shape. These yield marginal, population-level effect estimates comparing outcomes under shifted versus unshifted glucose distributions. Nuisance functions, i.e., the outcome regression and treatment mechanism, were estimated using the SuperLearner3 ensemble algorithm with five-fold cross-validation. TMLE relies on standard causal assumptions, including conditional exchangeability and positivity. We examined treatment feasibility across glucose strata and tabulated covariates by exposure to assess potential violations. Because TMLE provides population-average effects but does not yield insight into covariate-specific heterogeneity, additional modeling was pursued.

To evaluate glucose trajectories and explore whether patient characteristics modified the association between glycemic patterns and outcomes, we fitted joint longitudinal–survival models using the JMBayes2 package (version 0.4.5). This approach simultaneously links longitudinal biomarker trajectories with a time-to-event model. We estimated two linear mixed-effects submodels: one for SOFA scores and one for insulin dosing, with hourly glucose values and other confounders included as predictors. We incorporated random intercepts per admission SOFA value and per hour and random slopes for each patient to capture individual trajectories over time. The longitudinal processes were then linked to a Cox proportional hazards survival model for death or discharge, allowing a flexible, non-constant association between evolving markers and the event risk. All continuous covariates were mean-centered and modeled using natural cubic splines with five knots (see supplementary Table 2 for model specifications). Bayesian estimation was conducted via Markov chain Monte Carlo using five chains with 16,000 iterations each, including a burn-in of 4,000 iterations, and employing non-informative priors. Convergence was assessed through trace plots.
Given the exploratory, hypothesis-generating nature of this work, we did not compute p-values and instead report 95% confidence intervals (TMLE models) and 95% credible intervals (joint models)."

4. The data collection period is not mentioned in the paper. Please add it.

Reply: We added the data collection dates for MIMIC IV to the abstract. Further, we rephrased the pertinent sentence in the first paragraph of the methods section:
„Data was extracted during 2023 from the open-access and de-identified MIMIC-IV v2.2 (2008–2019) using Google's BigQuery software."

5. For the Results, Table 1 usually summarizes the data according to the primary outcome (in-hospital mortality in this paper).
Reply: We thank you for your comment. Table 1 is presented by exposure (diabetes status) rather than by outcome because, in accordance with standard epidemiologic and causal inference practice, it is intended to describe baseline covariates prior to the occurrence of in-hospital mortality. This is also in line with the CONSORT statement for RCTs and the STROBE statement for cohort studies. Please also refer to the STROBE checklist which we added to the supplements by request of the other reviewer. Stratifying by outcome would reflect post-exposure information and could introduce bias in descriptive comparisons.

6. Apart from time-weighted average glucose, glucose is a highly variable value. You may consider evaluating other variables like glycemic coefficient of variation.
Reply: Thank you for the suggestion. While it is true that glucose is highly variable, our analysis explicitly accounts for this variability. In the TMLE models, we used time-weighted average glucose to estimate population-level causal effects. However, in the joint modelling framework, we incorporated hourly glucose measurements as longitudinal predictors (see ***supplementary Table 5****), which captures both inter- and intra-patient variability. Therefore, additional summary metrics such as the glycemic coefficient of variation would not materially add information beyond what is already modeled.*
We highlighted this by changing the pertinent sentence in the discussion section as follows:

„While TMLE used time-weighted averages, joint modeling used the full hourly glucose series, capturing both within-patient variability and time-dependent confounding. The fact that both methods provided similar results, despite their conceptual and statistical differences strengthens our core finding that lowering glucose more aggressively did not meaningfully improve outcomes in septic ICU patients."

7. The study found a higher risk of hypoglycemia in patients with tighter glucose control. However, it did not address in detail how insulin dosing was managed or how adjustments might have contributed to hypoglycemic events.
Reply: We agree, this is a valid point. Unfortunately, we are not aware of any publicly available, detailed ICU insulin-management protocol from the Beth Israel Deaconess Medical Center (BIDMC). As a result, we cannot reconstruct how tightly treatment depicted in the data was adhering to the local internal standard. However, there is one study looking at BIDMC practices through utilization of MIMIC III data. We have now added a statement to the limitations section noting that lack of protocol-level data limits our ability to attribute hypoglycemia risk specifically to insulin dosing strategy rather than to unobserved management factors:

"Because detailed institutional insulin-infusion protocols and glucose-monitoring frequency from Beth Israel Deaconess Medical Center are not publicly available, we cannot exclude that variation in dosing adjustments, measurement intervals, or protocol adherence contributed to the observed hypoglycemic episodes. However, an analysis at aforementioned institution

found heterogenous insulin treatment strategies vary significantly, irrespective of blood glucose level or diabetic status.”

8. The conclusion in the abstract does not align well with the conclusion in the main body of the article. Please check it.
Reply: Thank you for pointing out this inconsistency. The conclusion now reads as follows:
„Through the combination of two innovative state-of-the-art statistical models, our data suggests a U-shaped association of glucose and mortality in the ICU setting, especially in non-diabetic sepsis patients, with an optimal average glucose between 160 to 190mg/dl. These results are in line with current guidelines recommending that intensivists should aim for a liberal glucose range in most patients. Employing target trial emulations in combination with causal inference in high-resolution healthcare data can provide meaningful insights into the relationship between treatment effects and patient outcomes. Even though the casual inference framework in an ICU setting should be viewed at as suggestive rather than definitive, the adoption of target trial emulations before planning prospective studies should become the norm as this methodology can help plan costly RCTs.”

9. The Discussion section should include an explanation for the negative results obtained in the study but simplify the discussion on the relationship between parenteral nutrition and changes in glucose or outcomes.
Reply: We agree with your assessment. We deleted the occurrence of parenteral nutrition where not really necessary. Further, we highlighted the negative results and added a short explanation.
The first paragraph of the discussion now reads as follows:

“Using a causal inference framework in a large cohort of critically ill patients with sepsis, we found no evidence that tighter glucose control with higher insulin exposure improved any clinically meaningful outcome. Across all TMLE analyses, glucose-lowering was not associated with reduced mortality, reduced SOFA burden, fewer nosocomial infections, or more hospital- or ICU-free days. Instead, lower glucose ranges consistently increased the risk of hypoglycemia. An average glucose between 160 to 190mg/dl seemed to be the optimal compromise. These findings were corroborated by joint modeling, which integrated hourly glucose values and patient-specific trajectories, but was not able to identify a covariate pattern or subgroup that benefited from lower glucose ranges…

… Interestingly, our analysis found that tighter glucose control was not associated with relevant improvement in clinically meaningful outcomes, neither for patients with or without diabetes, but conferred a higher risk of adverse outcomes such as hypoglycemia. Our negative results are likely explained by the fact that any potential benefit of lower glucose levels is counteracted by insulin-induced glycemic variability and hypoglycemia, both of which are independent predictors of mortality in sepsis . Moreover, stress hyperglycemia in sepsis may represent a partly adaptive metabolic response, particularly in non-diabetic patients, which could explain why lower glucose ranges did not translate into improved outcomes. This is in line with several previous studies, most recently with the study by Gunst et al. Our treatment ranges were also in the range of 140-180 mg/dl proposed by current guidelines.
…”
